# Case study on long-term deformation monitoring and numerical simulation of layered rock slopes on both sides of Wudongde dam reservoir area

**DOI:** 10.1038/s41598-024-57598-7

**Published:** 2024-03-22

**Authors:** Chen Ding, Kaixi Xue, Chaohui Zhou

**Affiliations:** 1 College of Civil and Transportation Engineering, Shenzhen University, Shenzhen, 518060 China; 2 School of Civil and Architecture Engineering, East China University of Technology, Nanchang, 330013 China; 3 Geotechnical Engineering Department, Nanjing Hydraulic Research Institute, Nanjing, 210024 China; 4 College of Civil and Transportation Engineering, Hohai University, Nanjing, 210098 China

**Keywords:** Slope deformation monitoring, High steep layered slope, Numerical analysis, Long-term stability, Engineering, Civil engineering

## Abstract

Layered rock slope exists widely. Because of its special slope structure, it is prone to bending deformation and toppling failure, which is a serious threat to engineering construction and safety operation. At present, the research of layered rock slope still has great innovation potential. During the construction of Wudongde Hydropower Station on Jinsha River, safety and stability problems such as slope geological structure development, face rock unloading and relaxation, and even slip and large deformation were encountered. Through field exploration, it is found that the rock and soil stratification of the slope on both sides of Wudongde Hydropower Station is highly obvious. At present, there is a lack of research on-site long-term displacement monitoring of layered rock high-steep slope, especially for layered slope in complex hydrogeology and construction environment. In order to strengthen the research on the deformation and stability of layered rock slope, this paper analyzes the measured displacement data of Wudongde hydropower station slope, and establishes three-dimensional geological finite element model with the help of numerical simulation software. The stability of the slope is calculated by combining the finite difference method and the strength reduction method. Finally, the evolution mechanism of the deformation of the layered rock slope is explained according to the geological structure characteristics. The main conclusions of this paper are as follows: the layered slope in the dam reservoir area is prone to deformation under the combined action of long-term construction disturbance and fissure water seepage, and the construction disturbance has a strong influence on the artificial excavation area below 1070 m, and the maximum rock mass deformation and surface displacement in the artificial excavation area of the slope reach 92.2 mm and 312.5 mm, respectively. However, the influence of construction disturbance on the natural mountain above 1070 m is limited, the valley deformation of the natural mountain on the left bank of the reservoir area is higher than that on the right bank, and the cumulative deformation is still less than 20 mm. The influence of seepage on the displacement of the area with higher elevation at the top of the slope is more obvious, and the influence of excavation and other disturbances on the displacement of the artificial excavation area with lower elevation is more obvious. The deformation of the river valley in the water cushion pond behind the dam increases slowly, and the change trend of the field deformation data is mostly consistent with that of the numerical calculation. The horizontal shrinkage of the mountains on both sides shows a contraction trend on the whole, and the maximum horizontal shrinkage calculated by numerical simulation is close to 20 mm, which is located at the elevation of 990 m.

## Introduction

The long-term stability of high and steep slopes on both sides of reservoirs has always been a research hotspot for scholars at home and abroad. The equilibrium state of the rock on both sides of the dam does not typically shift significantly immediately after dam impoundment, and the creep process of the rock and soil on both sides of the dam often takes a long time^1^, and the shift in fissure water pressure caused by the rising water level is the main driving force for the plastic deformation of the rock in the dam site area^2^. 1963 Vajont arch dam in Italy^3^ near the left bank of the reservoir area of a large area of landslide is the result of long-term creep of the soil by water pressure. The volume of the landslide body was as high as 250 million m^3^, and since the completion of the dam in 1959, the shore slope displacement was large and small with the reservoir storage and pumping, and various reinforcement measures were never able to fully eliminate the valleyward deformation, which eventually led to a huge disaster. According to the data given by Yang et al.^4^ on the post-convergence displacement of the deformation of the hills on both sides of different dam projects, during or after the completion of water storage, the valley deformation of Jinping dam is about 15–30 mm, Ertan about 3.5 mm, Xiluodu about 30–49 mm, Lijiaxia about 15–30 mm, and the valley deformation of Xiluodu arch dam shrinks significantly in the early stage of water storage. The Zeuzier hyperbolic arch dam^5,6^ was subjected to 75 mm valley contraction and deformed upstream to 125 mm. The Kolnbrein arch dam^7^ was damaged by a 100 m long horizontal tension crack in the heel area when the storage level rose from 1860 to 1890 m under the action of water pressure, and the grouting curtain was damaged. In Russia and France, the two famous Sayano-Shushenskaya arch dams^8^ and Tolla arch dam^9^, each with multiple cracks in the dam heel area at the beginning of water storage, have caused great concern in the engineering community. Cheng et al.^10^ scholars analyzed that the effective stress principle of fractured rock makes the yield surface of Vajont arch dam reservoir slope shift extremely, which leads to irreversible plastic deformation and damage, and is the main cause of reservoir bank hill damage and river valley shrinkage during initial water storage. Jiang et al.^11^ proposed a new energy index, local energy release rate (LERR), for the intense rock bursts encountered during the tunnel excavation of Jinping II hydropower station. This indicator helps to understand rock burst from the perspective of energy release and can better predict the intensity of rock burst and the depth of protrusion crater. The scholar also selected three engineering cases for validation, and the results show that the proposed new theory matches well with the actual rockburst results in terms of scale.

Case studies on slope stability are moderately abundant, and the methods used by scholars mainly involve displacement monitoring method^12–14^, model test method^15,16^, limit equilibrium method^17^, finite element method^18–22^ and geostatistical method^1,23,24^. On-site monitoring is a key means to study the slopes and mountains on both sides of the dam. Li et al.^25^ monitored the valley deformation during water storage in a high arch dam in China and found that the measured value of valley narrowing lateral deformation reached 89.54 mm as of October 2018. After geological investigation and numerical study, it was concluded that the valley deformation over time is caused by the rheological properties of the rock body in response to changes in hydrogeological conditions. The head of artesian flow in the deep pressurized aquifer increases after impoundment, and the mechanical parameters of faults and shear zones weaken, which in turn can cause horizontal valley deformation of the mountain, which is related to specific hydrogeological formations. Xu et al.^26^ studied valley deformation in steep arch dams and its relationship with water level fluctuations. Displacement and water level data monitored over a 5-year period were used to determine the possible relationship between displacement and water level. Valley deformation was found to be closely related to reservoir fill and drawdown cycles; comparison of valley displacement rates with reservoir levels indicated that the effect of reservoir lowering on valley deformation was greater. Yang et al.^27^ investigated the effect of dam filling and reservoir storage processes on valley deformation based on the Jinping No. 1 high arch dam in China. The results showed that the deformation of the river valley was contracted with or without considering the construction and storage processes. The regularity of the development of the valley deformation is mainly influenced by the water storage process, and the magnitude of the valley contraction is mainly controlled by the dam pouring process. The magnitude of deformation in the upstream valley is significantly larger than that in the downstream valley.

Gao et al.^28^ conducted a field case study on the slope project of the K5 + 220−K5 + 770 section of the TJ1A marker of the Jiangweng Expressway in Guizhou Province, China. The study aimed to address the challenging issues of slope support design and parameter selection for seepage analysis calculations. Five deep displacement monitoring points were established on the landslide section being studied. The potential slip surface of the slope was determined based on preliminary investigation and monitoring information. The analysis of the support process considered the influence of seepage. The superimposed pore water pressure was calculated using finite element simulation software to assess its impact on the deformation characteristics of the slope. The scholar analyzed the simulation parameters using depth displacement monitoring data and the *P*-value test method. They also used the pore water pressure-e superposition calculation method to analyze the stress characteristics of the slope support structure after applying steady-state seepage. The results showed that the new pile-anchor support structure had a significant effect on the original pile-anchor support structure^29^. Gao et al.^30^ also proposed a superposition calculation method of pore water pressure to realize slope stress-seepage coupling. The research results indicate that the most dangerous working condition during slope excavation and support is the secondary excavation. It is essential to note that the reference significance of extreme values and safety factors decreases when localized displacement and plastic strain concentration areas are present on the slope.

However, the research on layered rock slope still has large limitations. According to some published academic results, the integrity of layered slope is poor, and the coefficient of safety and groundwater seepage law are comparatively different from that of ordinary homogeneous slope, and even the shift rule of the stability of layered slopes in different stratification states will be different^14,16,31^. Although a large number of academic papers on in-situ monitoring of homogeneous slope displacement have been published, no scholars have conducted field case studies on the slopes on both sides of the reservoir area of a hydropower station under the condition of laminated rock mass. And numerous scientific problems remain to be explored, especially the destruction mechanism of high steep type slopes on both banks under the action of long-term excavation disturbance and change of reservoir water level, and intervalley contraction and deformation on both banks, which are some critical potential innovation points. On the basis of previous research results^32–36^ and relying on the Wudongde Hydropower Station monitoring project of Jinsha River, the research conducted a case study and numerical simulation of layered soil slope. This project is the fourth largest hydropower plant in China and the seventh largest in the world. According to the geological investigation report, the left bank of the spillway outlet is a typical layered rock-soil slope, and the layering state is a down-dip inclination. The left and right bank slopes of this hydropower project have experienced dumping deformation and even local instability damage during the construction process. Due to the importance of the Wudongde Hydropower Station, it is significant to follow up and analyze the deformation of the valley and the slope monitoring data. Furthermore, in order to evaluate the slope stability of Wudongde dam reservoir area more comprehensively, study the development law of valley shrinkage deformation, and determine the safety factor and the corresponding potential failure mode of the high steep slope on the left bank of the spillway tunnel. In this study, the safety factor calculation program of slope based on strength reduction method was prepared by using Fish language in FLAC^3D^ to execute numerical simulation.

## River valley and slope deformation monitoring analysis

### Engineering geological overview

**Figure 1:**
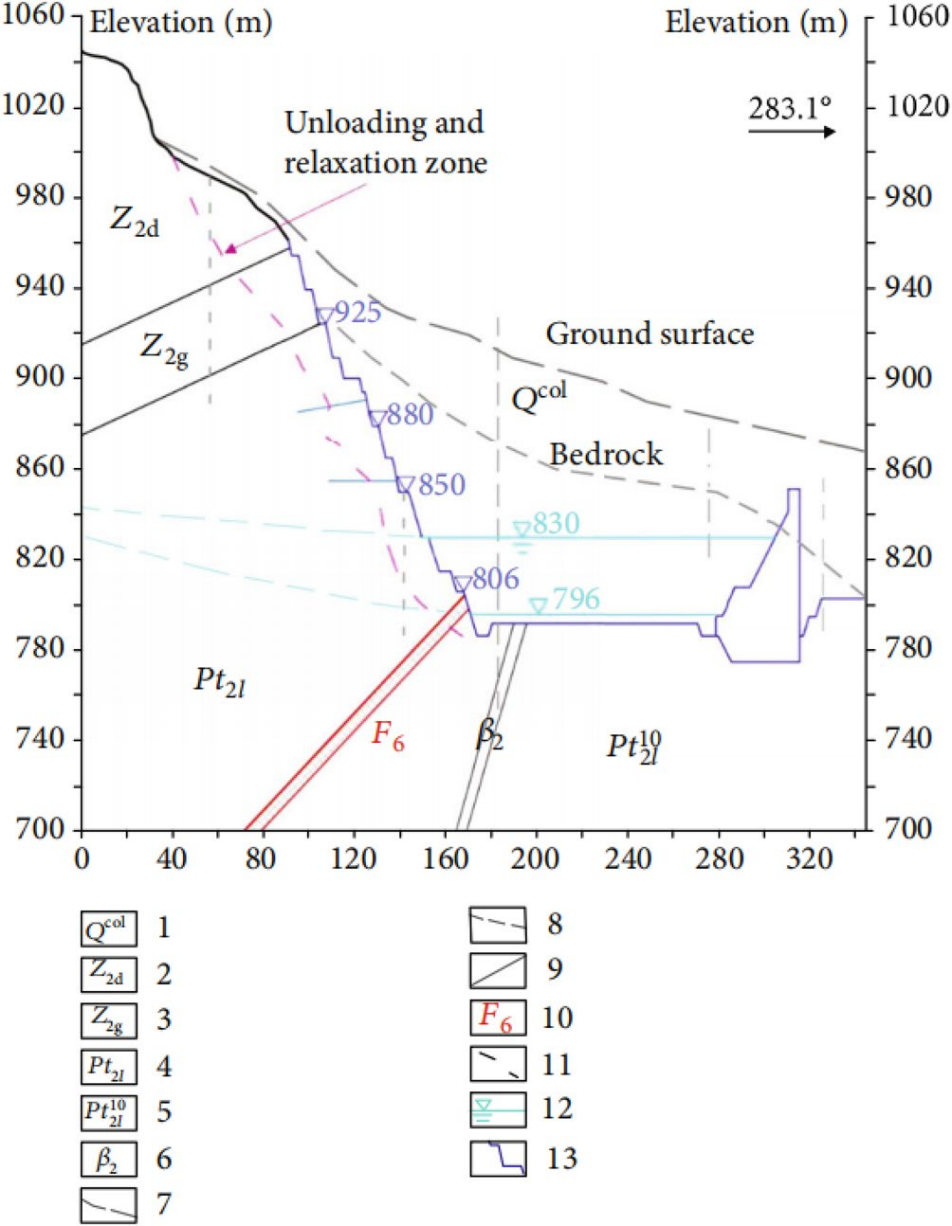
Typical engineering geological section of the left side slope. 1: collapse accumulation 2: gray to light gray thick gray dolomite of the Dengue Formation; 3: gray thin-layered dolomite sandwich and ultra-thin shale of the Guanyin Rock Formation; 4: gray thin-layered dolomite (specifically, the strata of the Falling Snow Formation downstream of the Huashangou Fault on the left bank); 5: gray rock with thin lime layer 6: gabbro 7: original topography 8: bedrock surface 9: lithologic boundary 10: faults and number; 11. Lower limit of artificial side slope slack zone 12: Groundwater table line 13: Design excavation line.

The part of the left side slope of the spillway outlet of Wudongde Hydropower Station with elevation greater than 1070 m is the natural exposed rocky slope side slope, with elevation 1070–1850 m as a steep slope, with an average inclination of 55°. The area below 1070 m elevation is an artificial excavation area, and the side slope before excavation is the gentle slope formed by Huaquan Gou deposit, with inclination of 10°–35°. The artificial side slope structure is transverse side slope, the highest elevation line is about 1070 m. The excavation elevation of the water cushion pond foundation corridor is 786.5 m, and the excavation height of the artificial side slope is about 183.5 m. The excavation of the first phase of the flood cave outlet started in June 2012 and ended in March 2014, mainly excavated to an elevation of 850 m. The excavation of the second phase started from March 2015 to May 2016, mainly excavated to the elevation of 830 m. The third phase excavation project from December 2016 to April 2017, mainly for the remaining part of the water cushion pond, tail channel and drainage channel, has been excavated to the bottom elevation 806 m, local excavation to elevation 796 m. In June 2017, the artificial side slope blasting excavation was completed, and the flood cave water cushion pond storage began. The typical excavation geologic profile of the slope on the left side of the spillway tunnel exit is shown in Fig. 1.

**Figure 2:**
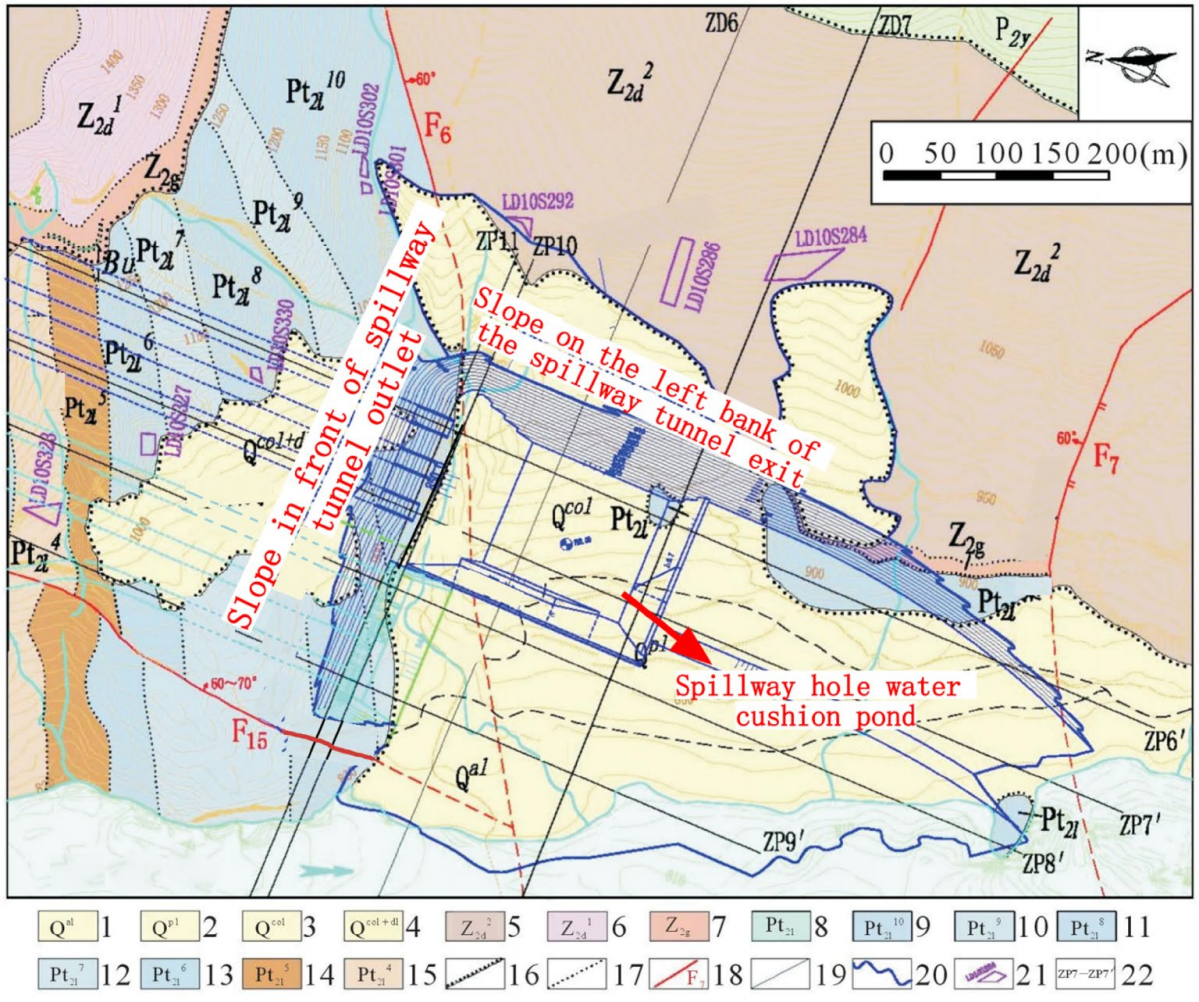
Geological diagram of slope on the left side of spillway tunnel exit. 1. Alluvial layer: 2. Diluvial layer: 3. Colluvial layer; 4. Slope collapse layer: 5. Dengying Group, Sect. 1:6. Dengying Group, Sect. 2; 7. Guanyinya Formation; 8. Snow Group (Left bank); 9. Paragraph 10 of the snowfall group; 10. Snow Group, paragraph 9; 11. Snow Group, paragraph 8; 12. Snow Group, paragraph 7; 13. Snow Group, paragraph 6; 14. Snow Group, para. 5; 15. Paragraph 4 of the snowfall group; 16. Unconformable stratigraphic boundary: 17. Lithologic boundary: 18. Geological fault and number: 19. Excavation line; 20. Opening line; 21. One thousand one level block and number; 22. Section lines and numbering.

The water cushion pool of the spillway is located in a high steep oblique layered stratum, and the artificial side slope to the left of the spillway outlet is located at the downstream side of the outlet of Huashan ditch, near the former Huashan ditch accumulation, and the geological conditions near the outlet of the spillway are shown in Fig. 2. The section of the geological section in Fig. 1 is approximately located at the ZD6 line in Fig. 2. The phenomenon of layering and blocking of the rock and soil body around the dam is serious, and each layered unit is loosely connected, and the gentle slope formed by Huashan ditch accumulation is below 1070 m on the left bank of the spillway, with a slope of 10°–35°. The natural exposed rock side slope are above 1070 m in elevation, and the steep slope at the top of the slope is above 1070–1760 m, with an average slope of about 55°. The natural slope of the engineering slope below the elevation of 1070 m and above it consists of sedimentary cover layers such as Z_2g_, Z_2d_, P_2y_, P_3em_, T_3bg_, J_1y_, J_2x_, etc. The stratigraphic direction is 340°–10°, dip E, inclination 28°–35°, i.e., the gently inclined left bank is upstream, and the angle between the stratigraphic direction and the slope direction is about 3°–37°, dip to the slope. The range elevation studied in this paper is between 796 and 1235 m.

**Figure 3:**
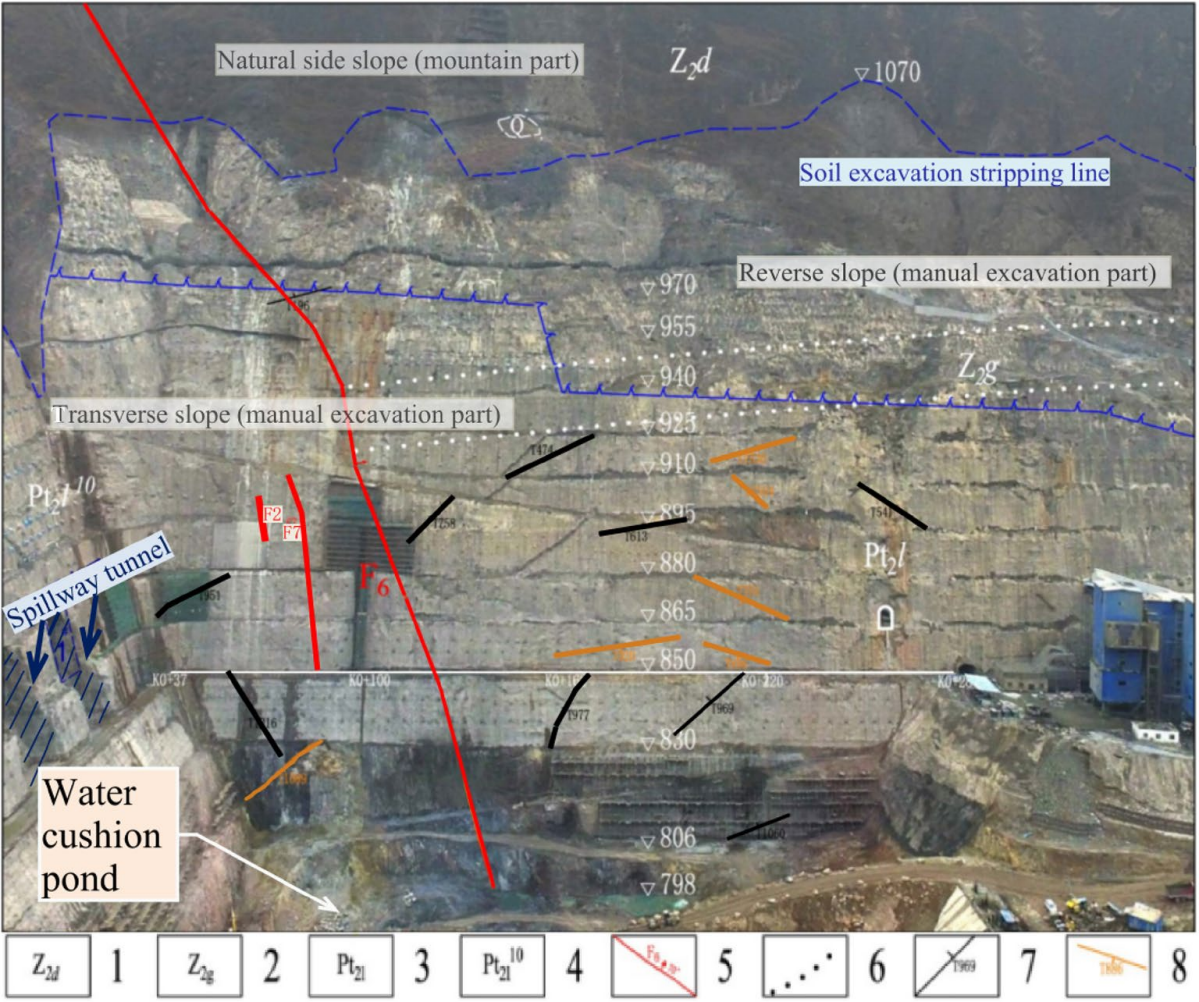
Distribution diagram of long and outward sloping cracks of slope on the left side of spillway tunnel outlet. 1. Dengying Formation grey–light grey thick layer dolomite; 2. Guanyin Rock Formation gray thin layer dolomite with thin layer and extremely thin layer shale; 3. Gray thin layer dolomite (specifically refers to the lower reaches of the Huashangou fault in the left bank of the Lixue Formation); 4. Gray, brick red interlayer with thin layer limestone; 5. Geological faults; 6. Lithologic boundary; 7. Cracks in incline slope; 8. Outside slope cracks.

The overall appearance and surface cracks of the slope on the left bank at the outlet of the spillway tunnel are shown in Fig. 3. The top of the artificial excavated side slope is reverse slope, the lower part is transverse slope, and the middle of the side slope is established with a sloping horse path, and the guide channel of the Huashan debris flow ditch is arranged at a later stage. The artificial side slope on the left side of the flood relief water cushion pond is a natural side slope (mountain part) with good stability. There are three more obvious faults: F6, F7 and F9. Among them, the large-scale fault is Huasan Gou fault F6 side slope is basically bounded by Huashan Gou fault F fault 6, and most of the rocks on the upstream side of the slope are slightly new. The downstream side is partly slightly new and partly weakly weathered. The fractures on the side slope are generally not developed, but locally developed, mainly occurring in plane fractures, and the length of fractures is generally 5–10 m. There are fewer fractures outside the slope, and there are only 6 fractures outside the slope with a length greater than 20 m, of which the longest is about 37 m. The fracture surface is generally straight and rough, mostly closed or slightly stretched, and with a calcium film or mud-calcium film. The horizontal depth of the original rock body weak unloading area is generally 8–15 m, and the horizontal depth of the micro-unloading area is generally 20–25 m. After excavation, the relaxation depth of the artificial slope is generally 17–28 m, of which the deepest is 29.2 m and the shallowest is 9.4 m.

**Figure 4:**
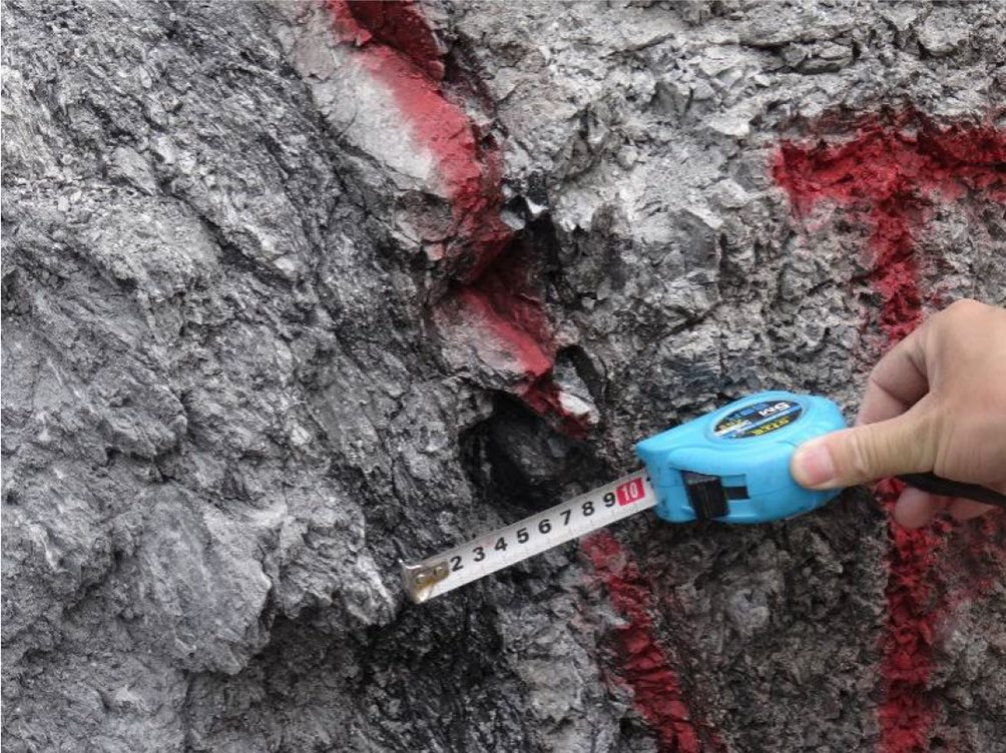
Rock cracks during slope excavation.

The main engineering geological defects exposed during the excavation of the slope included blockwork, fractures outside the slope, the Huashangu fault (F6), and poor rock masses. A total of 33 cracks were found on this slope, of which only 6 were rock deformation cracks (see Fig. 4), which were found in late April 2014 and mid-June 2016, respectively. These rock deformation cracks are all located in the local ridge-like prominence above the opening line downstream of the F6 fault, with rough fracture surface, extension length of about 10 m, crack opening width less than 4 cm, and depth less than 0.4 m, all extending into the subsurface rock mass. The causes are mainly related to the slope relaxation and deformation. The remaining 27 cracks are all shotcrete cracks, mainly distributed on the stripped slope and engineered slope surface near the F6 fault, with rough crack surfaces, visible extension lengths less than 12 m, crack opening widths less than 4 cm, and maximum visible depths less than 0.1 m. They are only exposed to the shotcrete, but do not extend into the rock mass. The reasons are mainly related to the shrinkage of concrete, temperature changes and other factors.

### Displacement monitoring equipment

**Figure 5:**
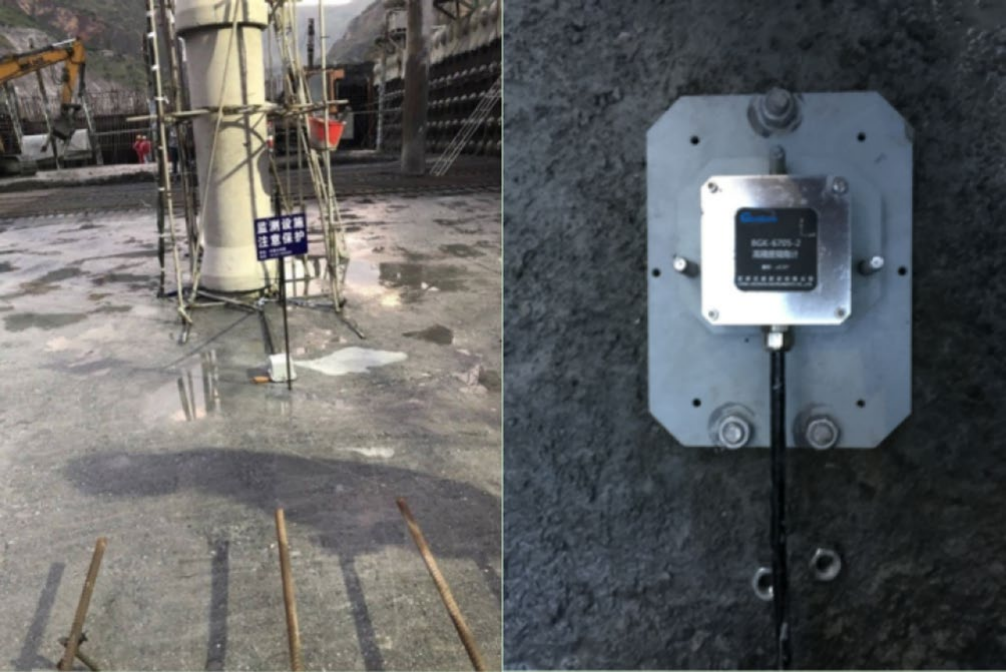
Displacement monitoring device.

During the construction of the left bank slope, there were two cases of steep increase in rock deformation and surface deformation, which aroused great concern. It is necessary to grasp the deformation of the slope and the force of the support structure during the period through on-site monitoring, effectively determine the deformation law and characteristics of the slope, analyze the factors that cause the change of the monitoring volume, so as to dynamically guide the program development, excavation construction and support. The project slope deformation monitoring adopts the multi-point displacement meter of model YT-DG-0600 series produced by Changsha Yito Monitoring Instruments Co., Ltd. Each set of equipment consists of multiple displacement meters, installation base, extension transfer rod, anchor head, etc. The resolution is 0.01 mm. The device has high accuracy and stability, and can be used for long-term observation by manual reading or automatic collection. The displacement meter shell of this project is made of stainless steel, waterproof and moisture-proof, and can resist high water pressure, and can be equipped with stainless steel mounting accessories, which can be used for long-term health monitoring under corrosive or other harsh environments, and the appearance of the displacement equipment is shown in Fig. 5.

As can be seen from Fig. 3, the mountain body on the left bank of the outlet of the spillway tunnel of this project is divided into natural slope and artificially excavated slope by dividing line. As the natural slope is formed under the action of natural geology, compared with the artificial excavation slope, it is less affected by excavation, blasting and other engineering activities. And the scale and mechanism of natural slope deformation may be quite different from that of artificially excavated slope. Therefore, the natural slope and artificial excavated slope are discussed and analyzed separately in this paper.

This project adopts a multi-point displacement meter composed of a multi-branch displacement meter, an installation base, a lengthened transfer rod and an anchor head, which are respectively installed in the parts of the mountains on both sides that need to be monitored to monitor the apparent displacement of the slope. the position of the monitoring point is appropriately adjusted according to the actual conditions of the site; the data are collected once every 30 days, and the transmission module connects the server to transmit the displacement monitoring data to the data acquisition center. Terminal data query, data analysis, when the deformation rate increases or abnormal changes, shorten the monitoring time interval, encrypt the monitoring times, find the deformation trend and fault in time, and formulate the corresponding disposal measures.

**Figure 6:**
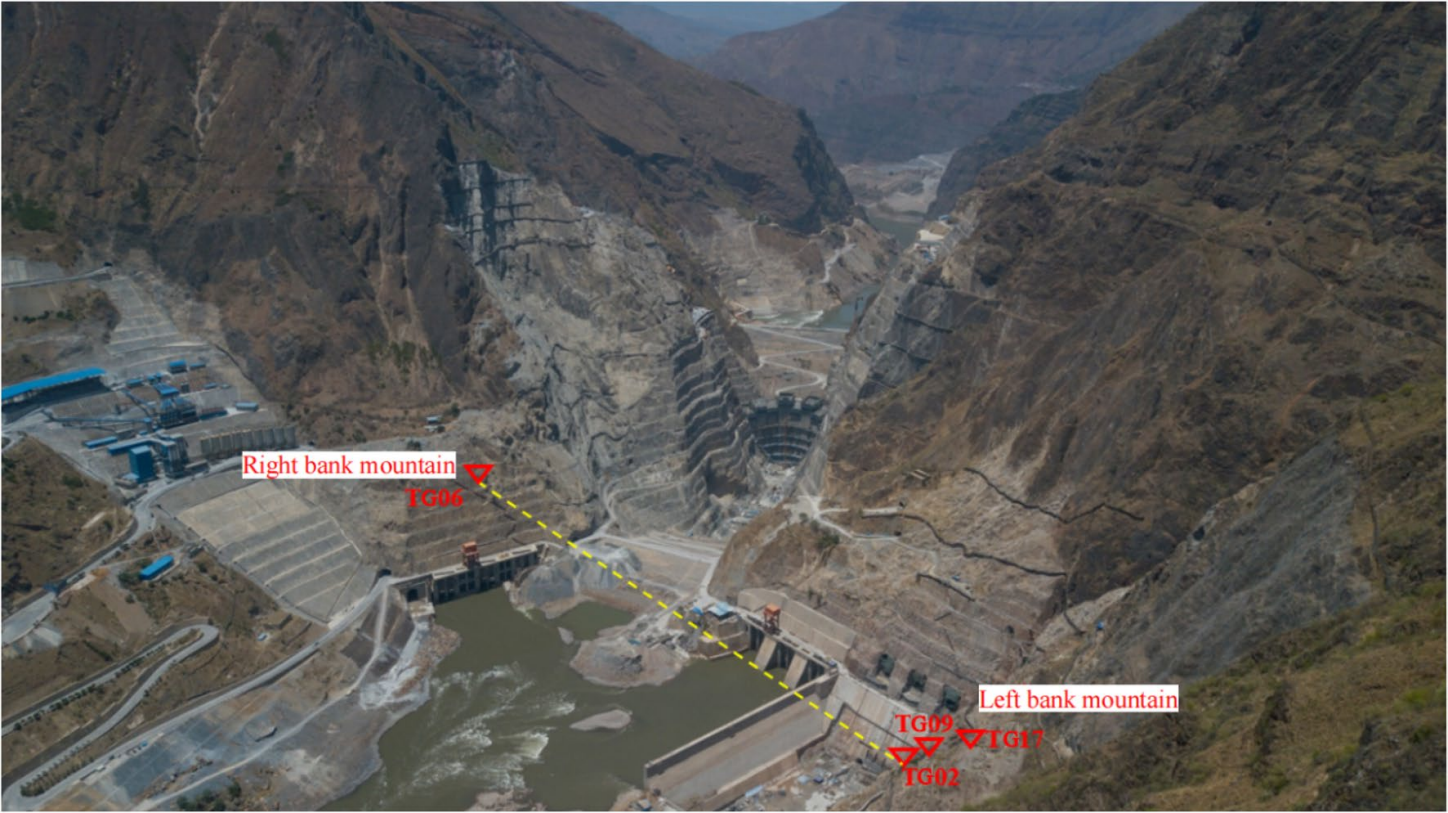
Distribution of mountain valley deformation monitoring points on both sides of water cushion pond in flood tunnel.

**Figure 7:**
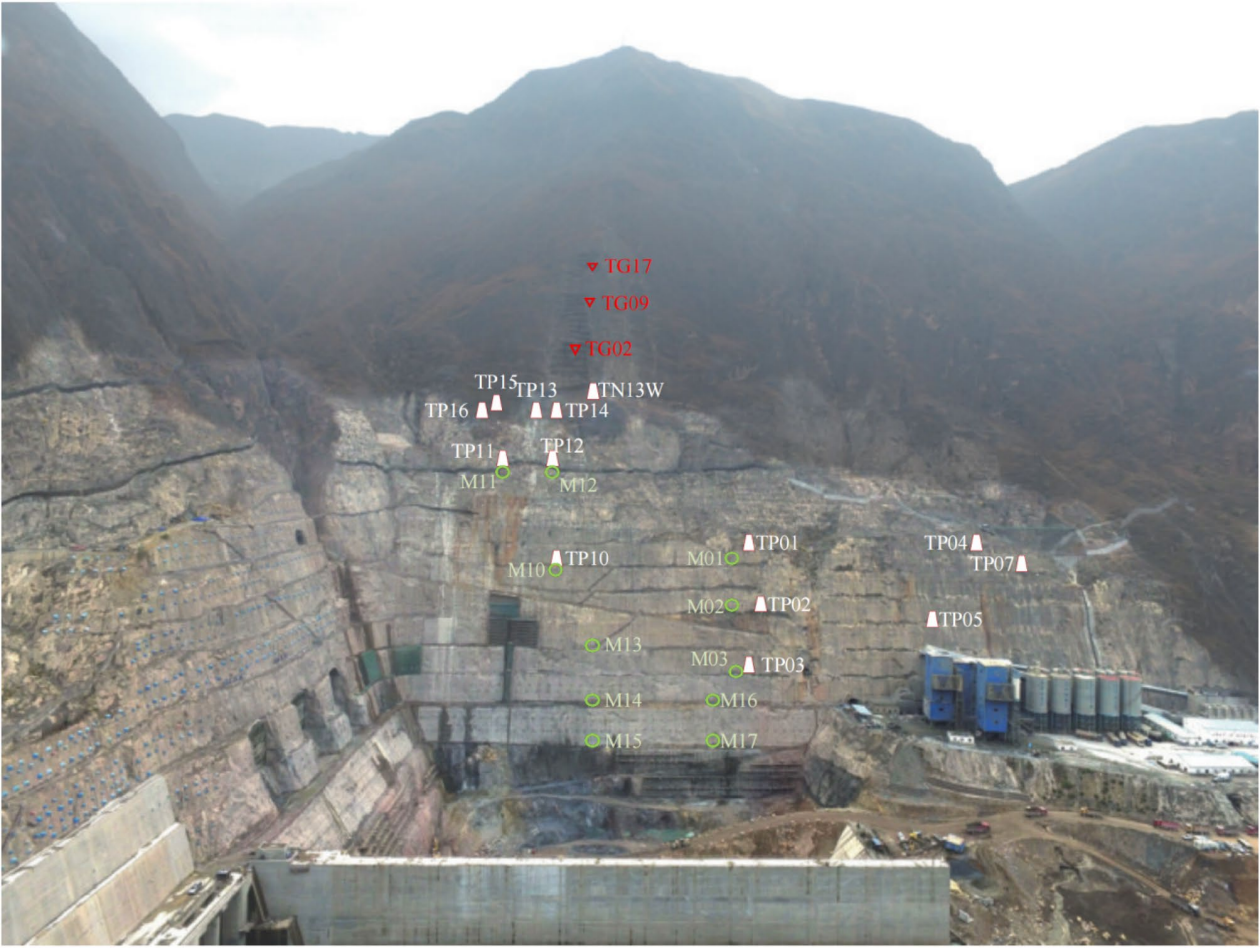
Distribution of effective monitoring points on the left bank slope.

While the Wudongde Hydropower Station is under construction, from June 2015 to March 2019, the construction unit established displacement monitoring points in the area with an elevation of more than 1070 m on both sides of the outlet of the spillway tunnel (for the unexcavated natural mountain part), numbered TG06, TG02, TG09 and TG17, respectively, to monitor the valley deformation in the reservoir area through the mountain displacement. The arrangement of the mountain displacement monitoring points on both sides of the spillway tunnel is as follows: the right bank elevation of the survey point TG06 is 1088 m, the left bank survey point TG02 is 1088 m, the left bank survey point TG09 is 1175 m, and the left bank survey point TG17 is 1235 m. Due to the different construction environment and conditions, the installation and recording time of the valley deformation monitoring points on the left and right sides are different. The data are recorded manually, and the recording interval is about 30 days. Affected by the site construction progress and other factors, the TG17 monitoring site was installed in 2015, and TG06, TG02 and TG09 were installed in 2016, so the time span of the data is inconsistent. The layout of monitoring points for the natural slope of the mountains on both sides of the Wudongde dam is shown in Fig. 6. Since 2013, six deformation monitoring sections have been arranged on the left side of the artificially excavated slope at the outlet of the spillway tunnel, 17 sets of multi-point displacement meters (numbered at the beginning of M) have been arranged and implemented to monitor rock mass deformation, and 16 apparent monitoring piers (numbered as TP or TN) have been arranged and implemented. Seventeen sets of borehole multi-point displacement meters are uniformly distributed in the lower reaches of F6 fault, among which 11 sets are arranged between F6 fault and the trailing edge slope of 850 m concrete system, and 6 sets are arranged in 850 concrete system trailing edge slope. Fourteen apparent monitoring piers are uniformly distributed in the lower reaches of F6 fault, 11 between F6 fault and the trailing edge slope of 850 m concrete system, and 3 between 850 concrete system trailing edge slope. However, due to the influence of on-site construction and other factors, as of July 2019, only 11 sets of multi-point displacement meters and 14 sets of apparent monitoring piers have been effective. The arrangement of effective monitoring points on the left bank slope of the Wudongde dam spillway tunnel is shown in Fig. 7.

### River valley deformation monitoring analysis

**Figure 8:**
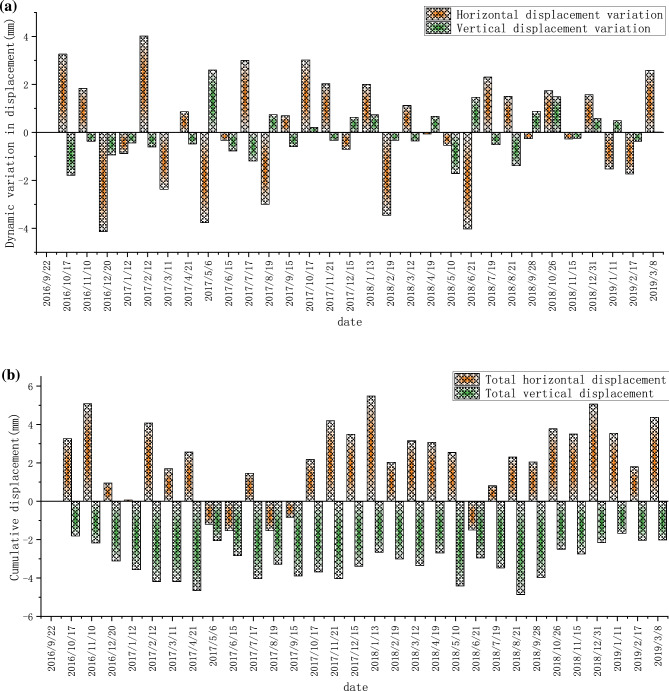
Data recording at the monitoring point TG06 located on the right bank: (**a**) Monthly displacement; (**b**) Monthly cumulative displacement.

Due to the overall displacement value of the slope on both sides of the spillway cave water cushion pool is small, the time interval of all the monitoring points to record data is about 30 days (1 month). The right bank was monitored from October 2016. The monthly dynamic variation and cumulative deformation of TG06 are shown in Fig. 8a,b, respectively, where positive horizontal displacement indicates deformation toward the valley frontage and positive vertical displacement indicates deformation toward the valley bottom. From the monitoring data, it can be seen that both the total horizontal displacement and the total vertical displacement fluctuate to some extent, but their trends are stable, and there is no obvious deformation in both horizontal and vertical directions of the right bank mountain, and the maximum accumulated deformation in the horizontal direction is about 5.5 mm, and the maximum accumulated deformation in the vertical direction is about 3.8 mm (toward the top of the mountain), and the monthly dynamic changes also mainly vary smoothly above and below the zero value.

**Figure 9:**
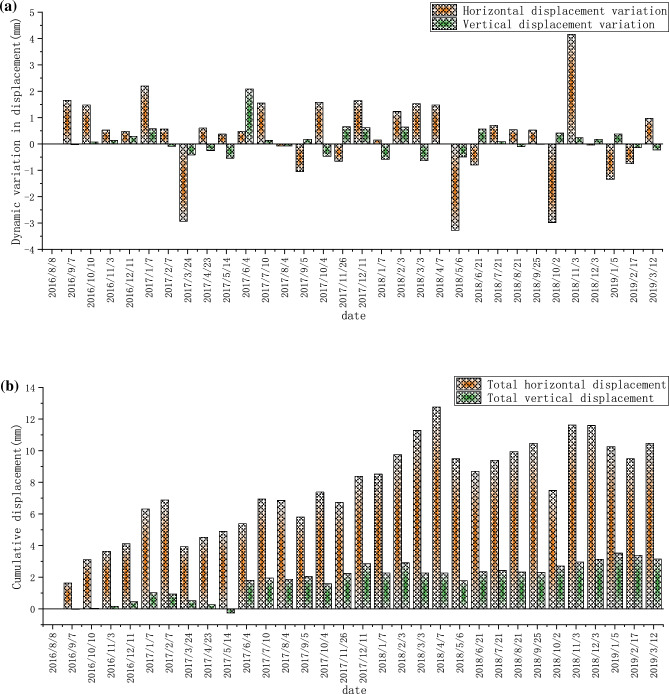
Data recording of TG02 monitoring point on the left bank: (**a**) Monthly displacement; (**b**) Monthly cumulative displacement.

**Figure 10:**
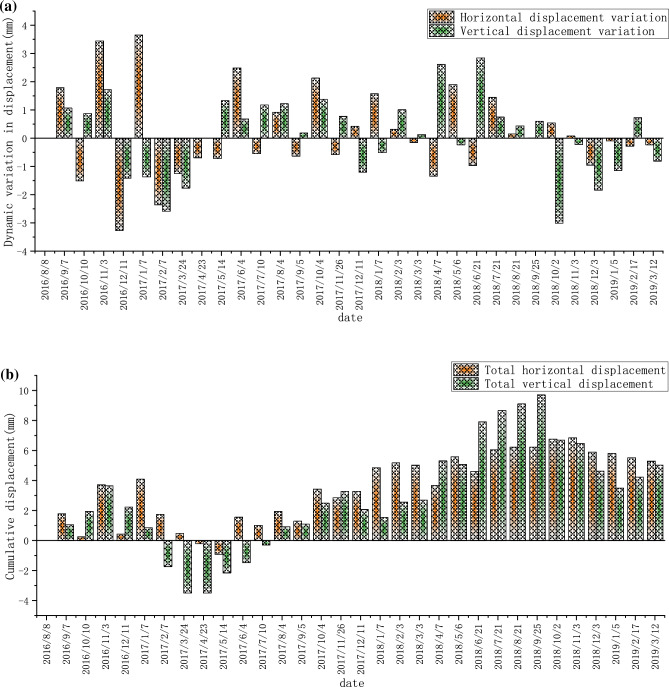
Data recording at the monitoring point TG09 on the left bank: (**a**) Monthly displacement; (**b**) Monthly cumulative displacement.

**Figure 11:**
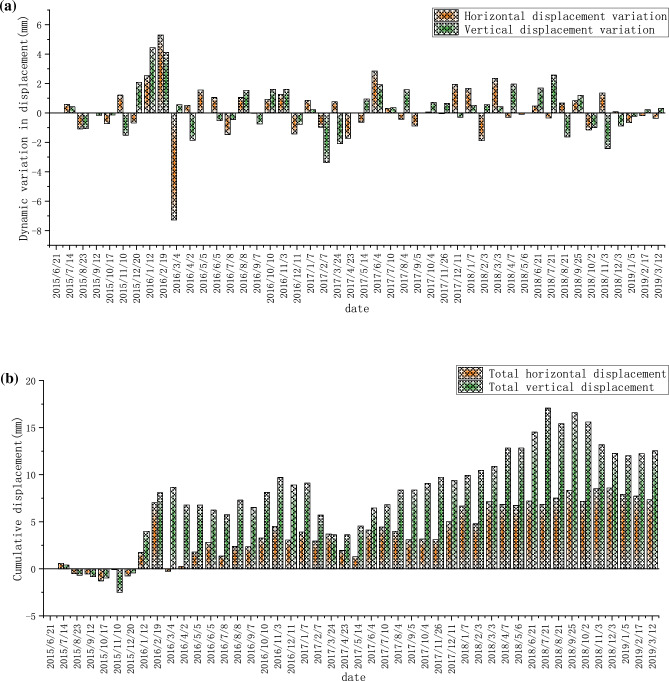
Data recording at the monitoring point TG17 located on the left bank: (**a**) Monthly displacement; (**b**) Monthly cumulative displacement.

**Figure 12:**
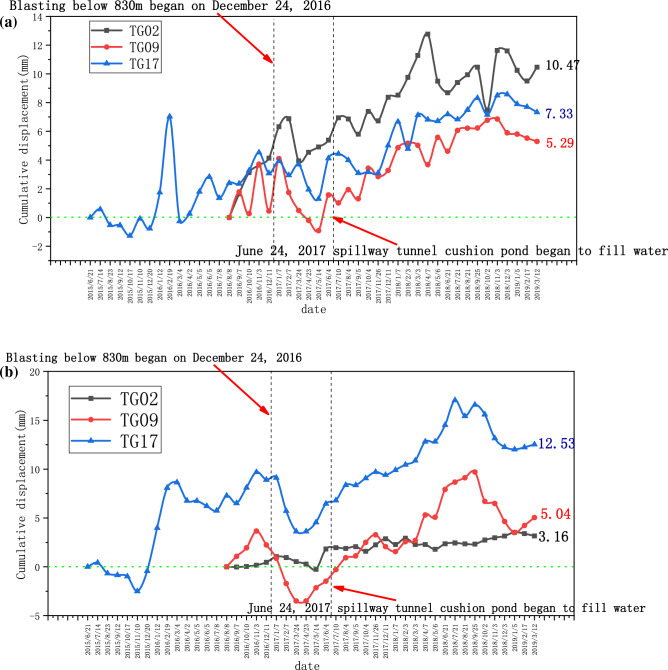
Comparison of cumulative displacement changes of TG02, TG09 and TG17 at monitoring points on the left bank: (**a**) Cumulative displacement in horizontal direction; (**b**) Vertical cumulative displacement.

The left bank has been monitored since June 2015, and the monthly dynamic and cumulative displacement data of TG02 are shown in Fig. 9a,b, respectively. The monthly dynamic displacement and cumulative displacement data of TG09 are shown in Fig. 10a,b, respectively. The monthly dynamic displacement and cumulative displacement data of TG17 are shown in Fig. 11a,b, respectively. Due to the complexity of the geological environment and the construction site, the displacement data of the three monitoring points do not reflect an obvious consistent response law, and whether in the horizontal or vertical direction, the occurrence time of the maximum displacement is different. The cumulative deformation of the three monitoring points on the left bank is summarized in one data chart, of which only TG17 is in normal operation from June 21, 2015 to September 7, 2016. The horizontal and vertical displacements are shown in Fig. 12a,b, respectively.

The accumulated deformation in the horizontal direction was most significant at the minimum elevation of TG02 (1088 m) and the accumulated deformation in the vertical direction was most significant at the maximum elevation of TG17 (1235 m). By observing the changes of horizontal and vertical cumulative deformation, it is found that after the water cushion pool of the spillway tunnel starts to fill with water, it has more influence on the deformation of the mountain than that of the blasting construction. For the horizontal direction, the cumulative deformation did not produce significant abrupt changes at the three monitoring sites after the blasting construction started on December 24, 2016; however, the cumulative deformation of G09 and G17 increased abruptly after the water filling started on June 24, 2017. For the cumulative deformation in vertical direction, the trend of abruptness increase after water filling is more obvious. This law corroborates with the laws expressed in other literature^37–39^, and a reasonable explanation is that the rise of water level causes the shift of fracture water pressure in the geotechnical body at the bottom of the slope, which in turn affects the stability of the bank slope. It is also noteworthy that the high and steep slopes in the study area exhibit significant stratification, while the seepage characteristics of the Layered geotechnical slope differ from those of the homogeneous slope. The integrity of the geological body will become poor after stratification, and the parting interfaces, joints, and fracture zones between different geotechnical units will easily become channels for groundwater and rainwater seepage from inducing landslides and collapses^16,40^. After the flood cave water cushion pond is filled with water, the high and steep layered slope may lead to a wide range of fluctuation of safety factor under the joint action of pond water, groundwater and rainwater, when the mountain is more prone to deformation. The law that the fluctuation of the water level of an impoundment aggravates the contraction and deformation of the valley has also been confirmed in additional engineering cases^26,27,41^. The famous Vajont dam landslide is entirely caused by the impoundment of the reservoir, which occurred in the third year after the impoundment of the reservoir^25^. However, some scholars^42^ proposed that when the valley amplitude deformation at the top of the water cushion pool is less than 50 mm, the safety of the seamless structure water cushion pool can be controlled, and water storage can reduce the influence of valley amplitude deformation on the adverse stress of the water cushion pool structure.

The total horizontal and total vertical displacements increase gradually and slowly with time, and the horizontal displacements are slightly larger than the vertical displacements in terms of increasing range and speed. Their monthly dynamic changes mainly fluctuate around the value of 0, and several large changes occur during this period. The comparison shows that both vertical and horizontal cumulative deformation on the left bank are significantly higher than that on the right bank, which is due to the fact that the slope on the left bank is closer to the flood relief tunnel and is more affected by the construction disturbance on site. However, although the deformation of the mountain on the left bank is larger, the horizontal cumulative deformation and vertical cumulative deformation of the three monitoring points are within 20 mm. The recording time of TG02 and TG06 displacement points is not consistent, therefore the value of the valley deformation cannot be obtained directly. But even if the maximum values of horizontal displacement and cumulative horizontal displacement of TG02 and TG06 are added up, they still do not exceed 20 mm. It is known that the long-term construction of the dam reservoir area has not caused obvious valley deformation, and the mountain body above the elevation of 1070 m on the left bank is basically in a stable state. Until June 2020, when the Wudongde hydropower station was formally put into operation for power generation, the four valley-oriented deformation monitoring points located on the natural rocky slope have all converged. Comparing with the measured deformation data of other water conservancy projects^4,43,44^, it can be seen that the disturbance of the mountain body in this project is limited, the support measures have effectively played a role, and the valiant deformation is properly controlled.

### Deformation monitoring analysis of artificially excavated slope

**Figure 13:**
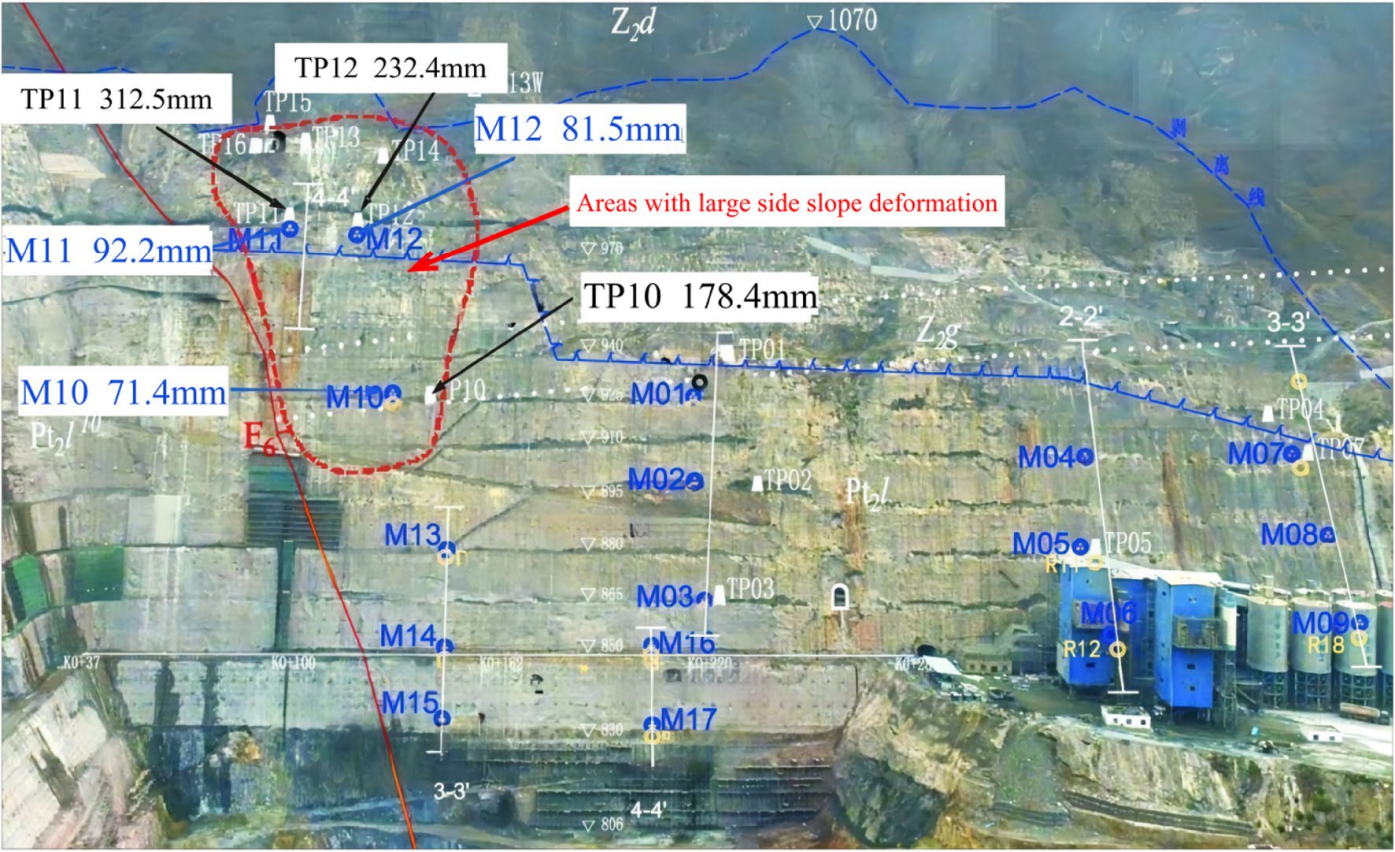
Left bank slope deformation area.

Figure 13 shows the area where the left bank slope shows large surface displacement and large rock deformation. All the multi-point displacement meter deformation monitoring data of this slope section have converged or apparently tended to converge since the blasting excavation was stopped on March 25, 2017.

**Figure 14:**
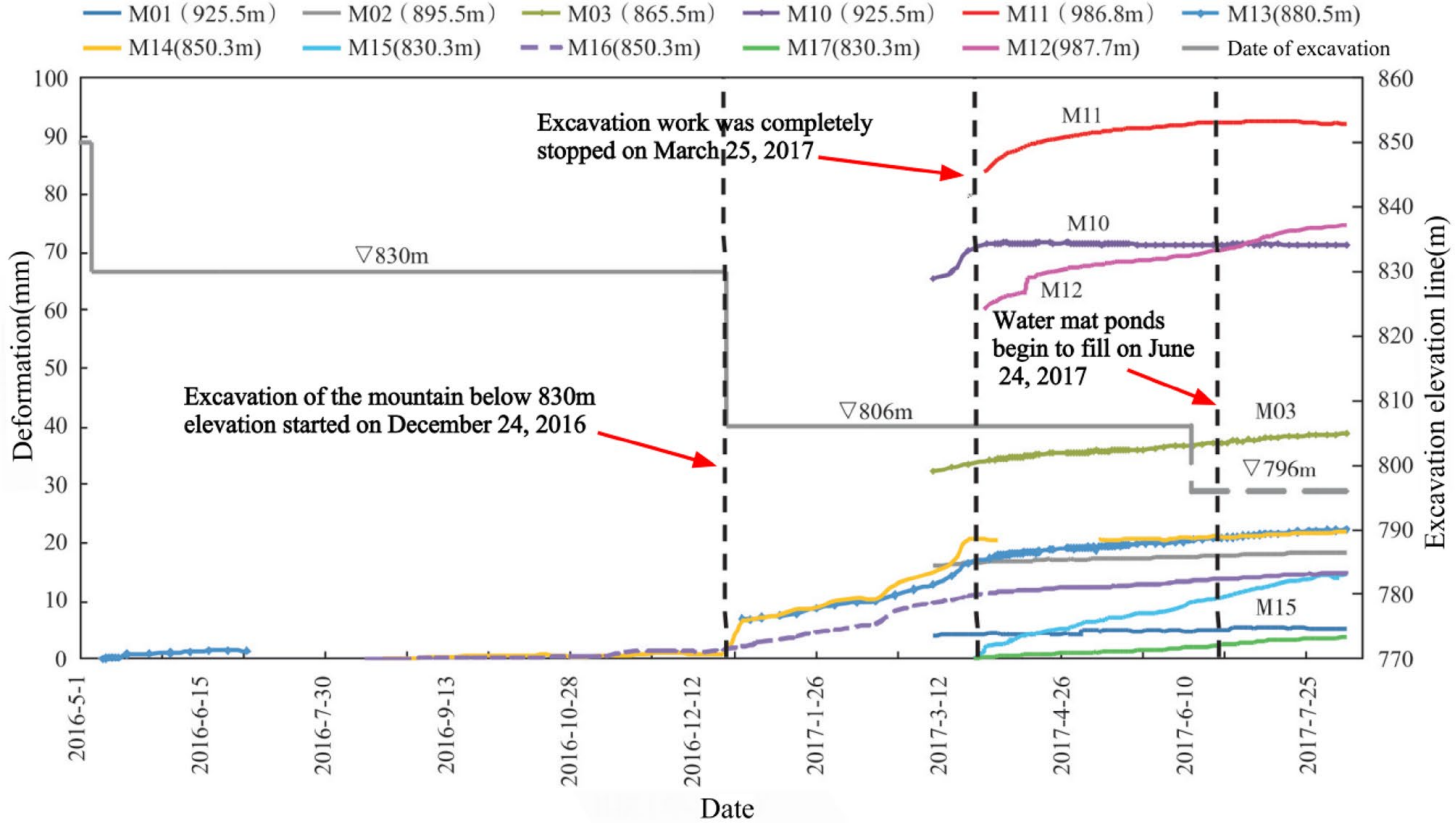
Relation curve between hole deformation and excavation process of multi-point displacement meter.

Since the artificial slope of the spillway tunnel was excavated gradually in layers, the first measurement time after the installation of each set of multi-point displacement meter was not consistent. The earliest time of the first measurement in the first phase was May 5, 2013, and there was also damage to the monitoring instruments. In order to make the image more intuitive, this paper only analyzes the cumulative displacement data from May 1, 2016 to August 10, 2017, as shown in Fig. 14. The monitoring depth is generally 30 m, and partly 40 m. Due to the influence of site construction and other factors, several sets of multi-point displacement meter monitoring data were missing from May 2015 to March 2017, and only M13, M14 and M16 captured a relatively complete deformation process. The deformation rate was essentially unchanged after the outlet water cushion pond was filled with water on June 24, 2017. The parts with larger local deformation monitored by point displacement meter (M10–M12) are mainly located in phase I 4–4 section on the downstream side of the F6 fault, which is adjacent to the F6 fault and located in the range of elevation 925.0–985.0 m on the downstream side of the F6 section, near the open line stripping slope ridge-like terrain, and the larger surface deformation is related to this fault and poor lithology, caused by excavation construction during the construction period. As of July 2018, among the 17 sets of multi-point rock borehole displacement meters, 13 sets have minor cumulative deformation at the orifice, below 25 mm, accounting for 76.5%; three sets have larger deformation, above 70 mm, with a maximum of 92.15 mm, for point M11. The points with larger deformation are located at the top of the protruding ridge-like terrain near the opening line, followed by the middle area of the slope, and the cumulative deformation of the slope behind the concrete system at elevation 850 m and other areas of the slope is slight.

**Figure 15:**
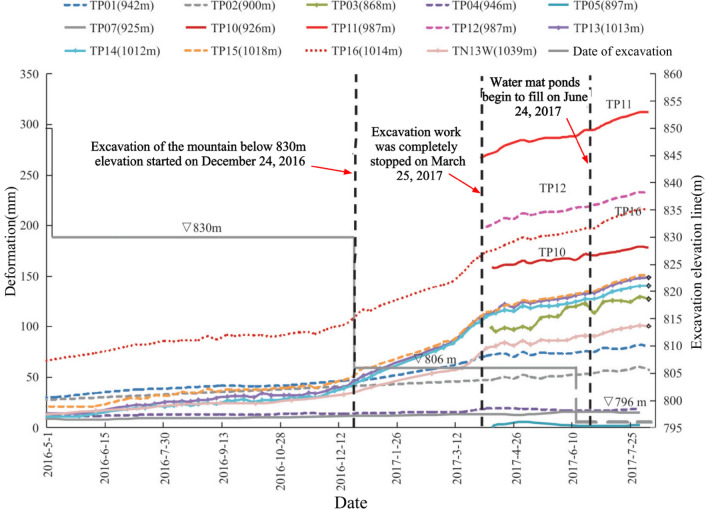
Relationship between total deformation of surface monitoring and excavation process.

The first measurement of each apparent monitoring point after installation was as early as September 16, 2013, and due to the influence of site construction and other factors, several groups of apparent monitoring data were missing for a long time from April 2015 to April 2017, only TP01, TP04, TP13–TP16 and TN13W captured a more complete deformation process, and the accumulated deformation of each monitoring point is shown in Fig. 15. According to the characteristics of surface deformation components, the slope is mainly found to be horizontal deformation and vertical settlement, mainly horizontal deformation. As of August 10, 2017, among the accumulated deformation components, the cumulative deformation in the direction of the horizontal plane is larger, generally up to 50–140 mm, and the maximum is 223.75 mm (TP11). The vertical settlement is slightly smaller, generally 14–80 mm, the maximum 214.5 mm (TP11); the cumulative deformation in the downstream direction is the smallest, generally 8–23 mm, and the maximum is 54.6 mm(TP03).

After blasting and excavation of the slope below elevation 830.0 m on December 24, 2016, the deformation fraction and total deformation of each group of apparent monitoring appeared to increase steeply and the deformation rate accelerated; after March 25, 2017, blasting and excavation were stopped and the deformation rate decreased significantly, and as of August 10, 2017, five of the 14 apparent monitoring points had converged, and the deformation rate of most measurement points had decreased significantly and tended to converge. In addition, according to Figs. 14 and 15, the area of larger surface deformation of the slope is consistent with the area of larger deformation of the rock mass, and the area of larger surface deformation is mainly located in the range of elevation 925.0–985 m on the downstream side of the F6 section (see the red circle in Fig. 13), which is adjacent to the F6 fault and located near the ridge-like terrain of the stripped slope of the opening line, and the larger surface deformation is related to the fault and poor lithology, which is caused by excavation caused by excavation.

The deformation of the left side of the excavated slope can be divided into 3 stages, i.e., the deformation intensifies, the deformation tends to slow down, and the deformation converges or tends to converge. From December 2016 to March 2017, the left side slope was excavated with high intensity blasting below 830 m in elevation, and the deformation volume and deformation rate of the slope were large, and the deformation of the slope gradually converged or seemingly tended to converge after the flood cave water cushion pond was filled with water. As of July 2018, the deformation of each monitoring point of the left side slope of the spillway tunnel outlet is in the convergence stage.

Meanwhile, comparing the deformation data of artificial excavation slope monitoring points and mountain deformation monitoring points, it can be seen that the artificial slope with elevation below 1070m is directly affected by blasting, excavation and mechanical disturbance, and the deformation is larger. The mountain body with elevation above 1070 m is more evidently affected by water filling of water cushion pond than construction disturbance, and the deformation of the mountain body in the valley direction increases more clearly after water filling. Referring to the field monitoring data of Jinping No. 1 Hydropower Station^45,46^ and Xiluodu Hydropower Station^41^, it is known that the initial shrinkage deformation of the slope on both sides of the dam is mainly caused by the unloading rebound of the rock mass after excavation, and in the later stage, with the impoundment of the reservoir area, the deformation will gradually converge.

## Numerical analysis of slope stability

### Constitutive equation

Based on the preliminary engineering survey data, and then the relevant initial parameters obtained through the indoor geotechnical test, the test process is carried out according to the relevant provisions of the current Chinese official published "Engineering Rock Test Method Standard" GB/T 50266. In this paper, the stability is effectively calculated using the built-in strength reduction equation in FLAC^3D^, where the parameter strength reduction is calculated with reference to formula (1) and formula (2):

1 $$\mathrm{c^{\prime}}={\text{c}}/{\text{F}}$$

2 $$ \varphi = \arctan \left( {\frac{\tan \varphi }{{\text{F}}}} \right) $$

where F is the discount factor of strength parameter; c, φ are the effective cohesion and internal friction angle, respectively; c′, φ′ are the discounted effective cohesion and internal friction angle, respectively.

The material intrinsic model is an isotropic elastic–plastic model using the Mohr–Coulomb (M–C) criterion, and its mechanical model is shown in formula (3):

3 $$ f = \sigma_{1} - \frac{1 + \sin \varphi }{{1 - \sin \varphi }}\sigma_{3} - \frac{2c\cos \varphi }{{1 - \sin \varphi }} $$

where, σ_1_ and σ_3_ are the maximum and minimum principal stress, respectively; f is the yield function.

The shear failure criterion is:

1. f > 0, plastic flow state;
2. f < 0, elastic deformation state;
3. f = 0, critical state of elasticity and plasticity.

The above is the shear damage criterion, tensile damage criterion for formula (4):

4 $$ f^{t} = \sigma_{t} - \sigma_{3} = 0 $$

where, σ_t_ is the tensile strength of rock mass.

In the finite difference procedure, the bulk modulus and shear modulus of the rock mass are calculated by formula (5) and formula (6), respectively:

5 $$ K = \frac{E}{{3\left( {1 - 2\nu } \right)}} $$

6 $$ G = \frac{E}{{3\left( {1 + 2\nu } \right)}} $$

where, K and G represent the bulk modulus and shear modulus of the materials used in the model, respectively; E is the elastic modulus of soil; ν is Poisson's ratio of soil.

### Numerical analysis and calculation

According to the quality classification method in the Rock Engineering Classification Standard (GB/T 50218-2014), combined with the actual characteristics of the rock bodies around the Wudongde Hydropower Station, detailed geological macroscopic analysis, classification and testing of relevant mechanical parameters were carried out on various rock bodies in the dam site area. After repeated analysis, the quality standards were finally determined. On the basis of the quality classification criteria of the rock bodies in the dam area, the quality classification of the studied engineering slope was further refined based on the lithology, rock structure and acoustic characteristics found in the current excavation. According to the detailed analysis results, its overall quality is of poor type, mainly IV1 to IV2, with a small amount of III2 and III1. Among them, the local debris in the fault zone is of grade V. The percentage of rock exposure area of the artificial slope at all levels is 0.4% for III1 rock, 6.1% for III2 rock, 59.6% for IV1 rock, 32.6% for IV2 rock, and 1.3% for IV2 to V rock of the fault F6 structure.

#### Numerical simulation solving process

The numerical model involved in this paper simulates the period of manual excavation to the final design elevation, which corresponds to the time of June 2017, when the blasting and excavation of the manual slope ended and the water cushion pool storage of the spillway began. The water cushion pool is finally excavated to the bottom elevation of 806 m, and the local excavation is excavated to an elevation of 796 m. At this time, the slope gradient is the highest, and the risk of slope instability is the greatest. Larger slope and heights will increase the deadweight of the slope and cause larger sliding forces. If the stability coefficient of the steepest phase of the slope under gravity can meet the safety requirement, then the slope of the previous excavation period can also meet the safety requirement.

**Figure 16:**
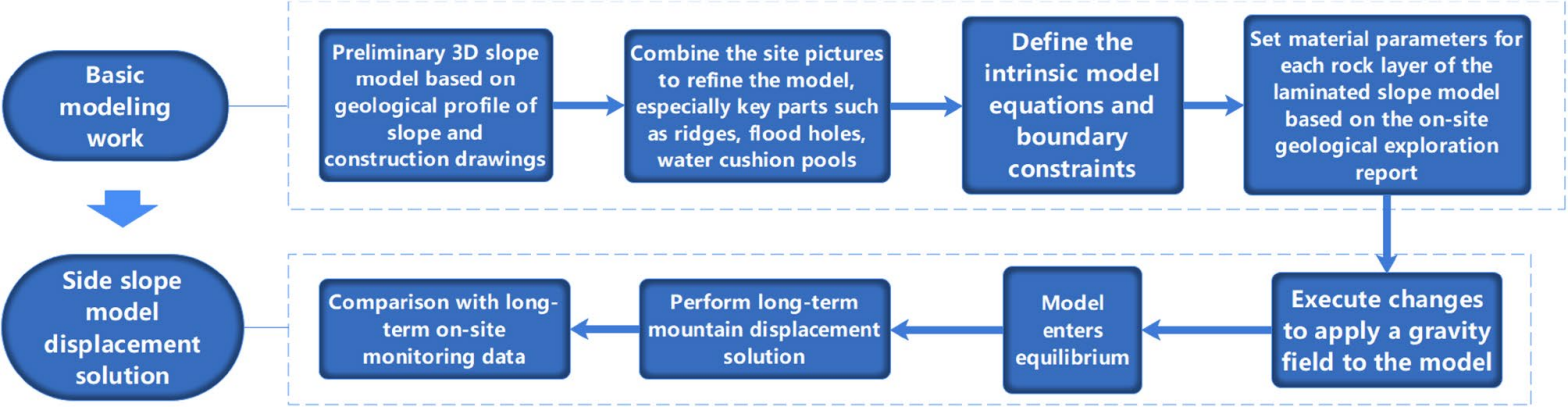
Numerical simulation solution flow of Layered slope.

Due to the large range of the selected x, y, z directions, it was set strictly according to the actual dimensions of the project. The outer boundary is constrained by fixed displacement, and the finite difference program FLAC^3D^ module is used to numerically simulate the large deformation of the rock mass under the gravity field and the tensile damage and shear damage along the face and rock mass. For the layered rock mass, the elastoplastic level model is used all over the nodal level. This intrinsic model can simulate the shear damage and tensile damage along the level and the rock mass, taking into account the anisotropy of the strength of the rock mass in both parallel and perpendicular level directions, which is beneficial to analyze the influence of the slope structure on the stability of the slope. Meanwhile, in the modeling process, the actual material structure is mainly simulated according to the polyhedral unit structure, the yield state of the material under the action of external forces is simulated by linear model or nonlinear intrinsic model, and the plastic damage and plastic flow of geotechnical materials are simulated by the "hybrid discrete method". In this paper, the general flow of the finite difference method is shown in Fig. 16.

**Figure 17:**
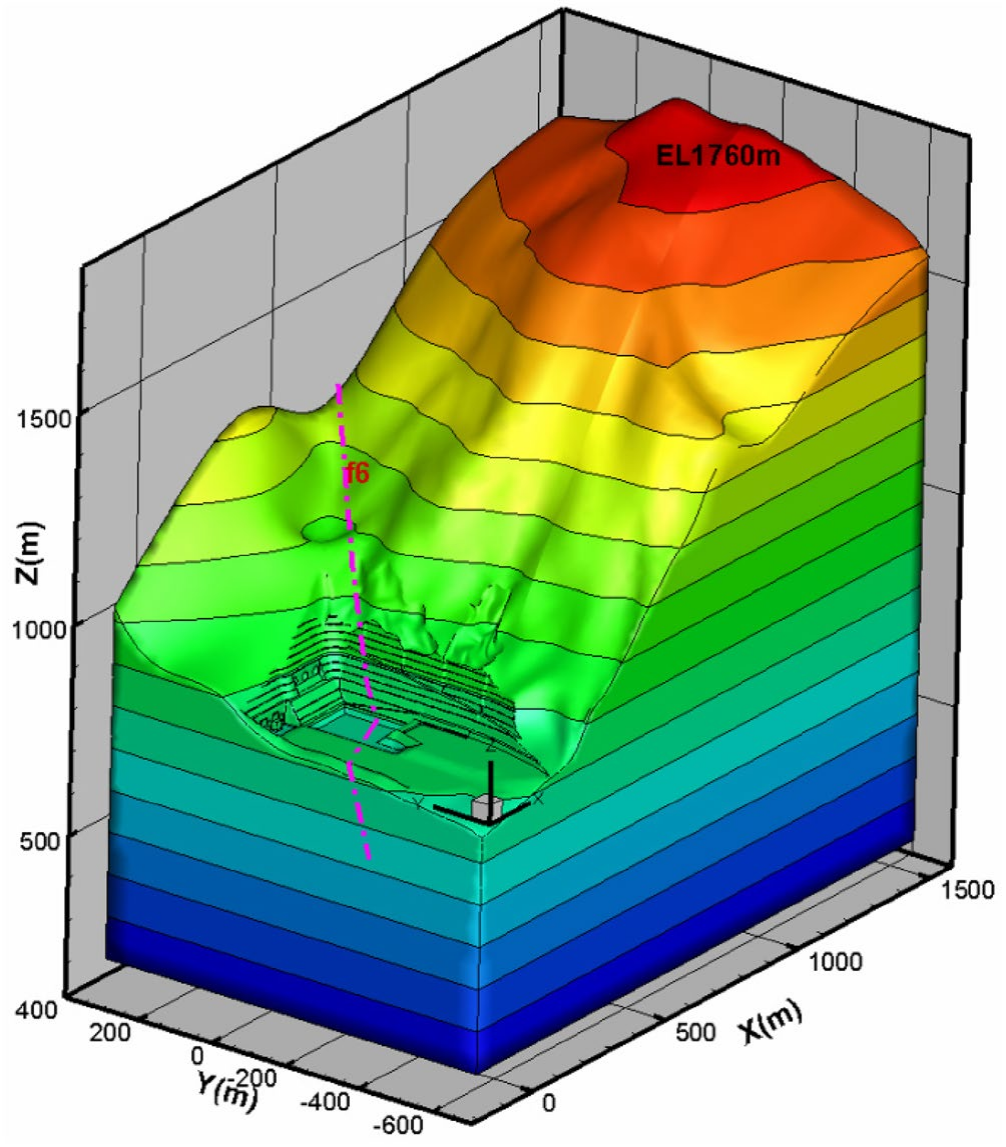
Three-dimensional numerical model of the left bank slope of the spillway tunnel.

Due to the deformation generated by self-weight, long-term creep, and excavation, rebound deformation, i.e. deformation toward the river valley, will occur. Taking the slope with large deformation (mountains on the left) as the object of study, which is located at the left hand side of the exit of the spillway tunnel, a three-dimensional layered geological generalized model of the whole left bank slope was established by FLAC^3D^ software according to the preliminary detailed engineering survey, combined with topographic and geological data, and the numerical model of the whole hill is shown in Fig. 17, and the boundary conditions of the model are set: three-way constraint at the bottom, horizontal constraint on several sides of the model directional constraints. After the model is established, the self-weight condition (i.e., the applied gravity field) will produce deformation to simulate the valley deformation (horizontal direction) generated by the slope in the actual project. If there is rainfall and earthquake on the applied physical field, of course, they will also affect the model deformation, but this situation needs to be analyzed additionally and are not considered in this paper. This paper considers the natural self-weight deformation and excavation rebound deformation under the action of long-term gravity field. Combined with the displacement safety monitoring data, the deformation damage mechanism and potential instability mode of the slope are calculated by using the finite difference method.

#### Stability analysis of the exit slope of the flood relief tunnel

**Figure 18:**
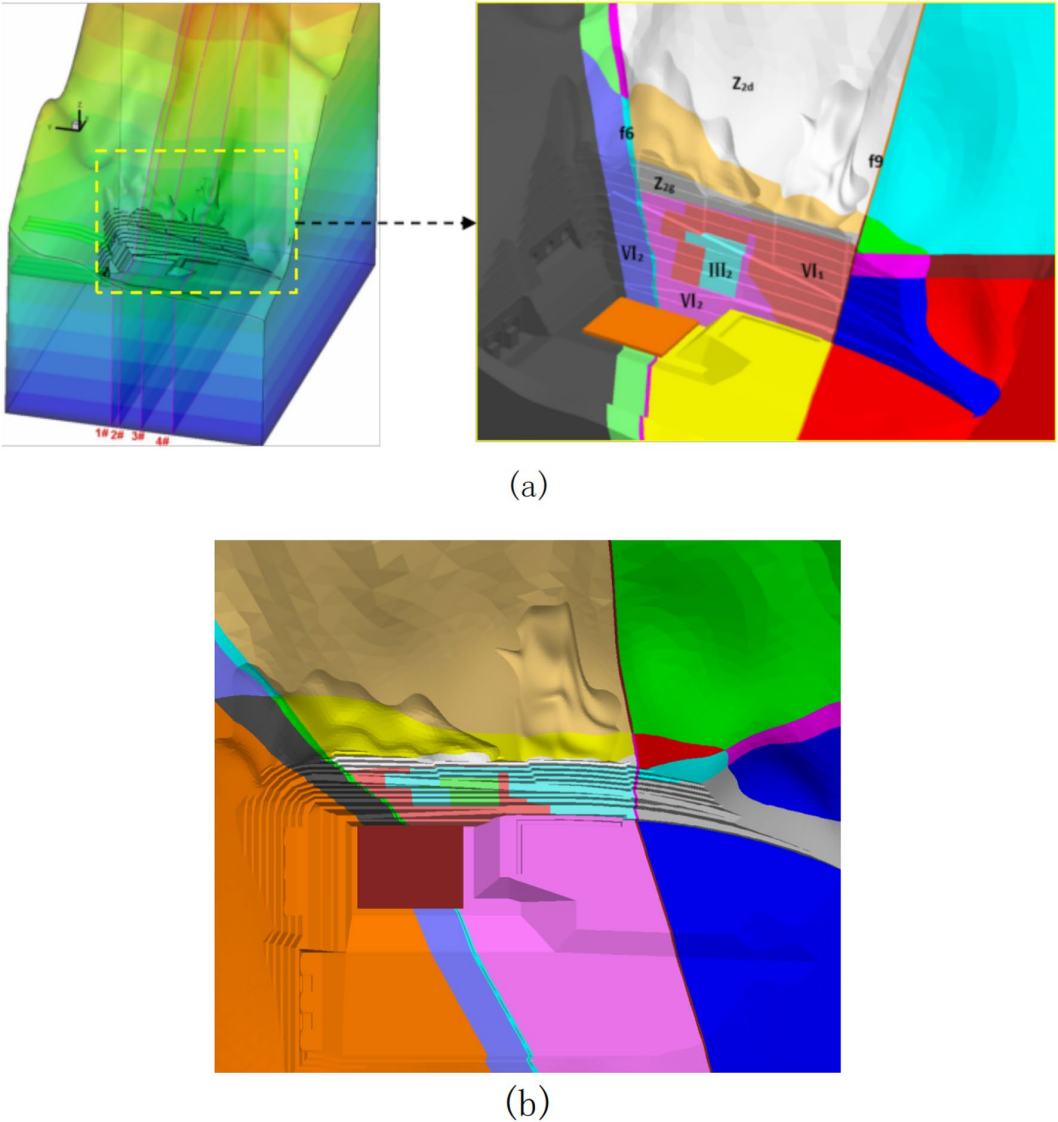
Numerical calculation model of the slope on the left bank of the spillway tunnel: (**a**) Each rock formation on the left bank of the water cushion pool of the spillway tunnel; (**b**) Top view of calculation model of water cushion pool in spillway tunnel.

**Figure 19:**
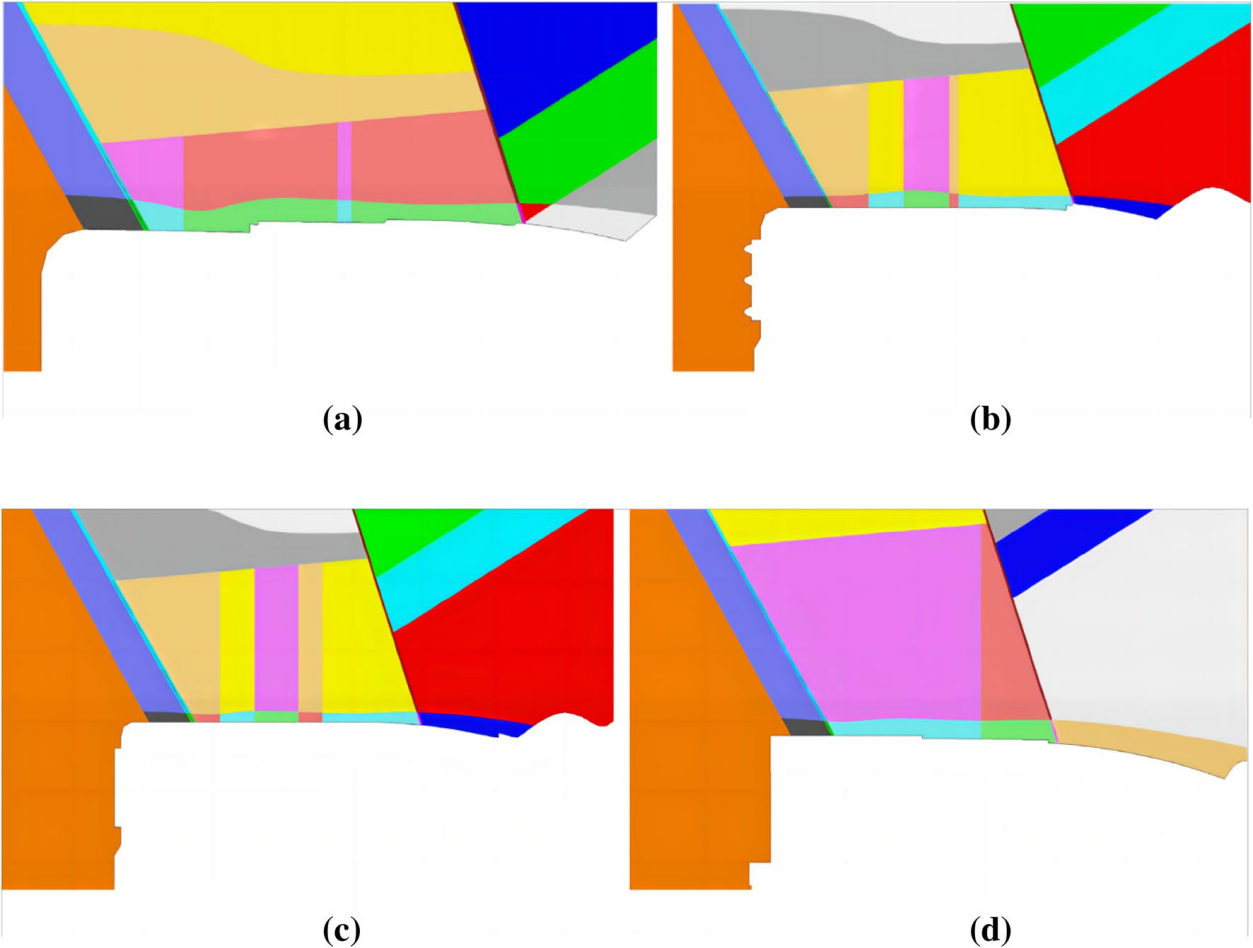
Horizontal section diagram of numerical calculation model of layered slope: (**a**) Elevation 895m; (**b**) Elevation 865m; (**c**) Elevation 850m; (**d**) Elevation 828m.

The rock and soil samples were obtained by in-situ drilling at the slope site, and then the mechanical parameters and lithology of different soil layers were tested by geotechnical tests. The mechanical parameters of various rock masses of the slope are given in Table 1, and Fig. 18a,b together show the appearance of the three-dimensional numerical model of the left bank slope of the spillway water cushion pool. It is perpendicular to the slope, where the x-axis is positive with the vertical side slope pointing to the inner side of the mountain, the y-axis is positive with the parallel side slope pointing to the upstream side, and the z-axis is positive with the lead upward (according to the right-hand system coordinate system), and the selected distances in three different directions (x, y, and z) in the dam site area are 1650 m, 1000 m, and 1550 m. In the calculation domain, Pt_21_^10^, P_t_^21^, Z_2d_, Z_2g_, Flower Hill Gully fault F6, fault F9, P_2y_, P_3em_, T_3bg_, J_1y_, J_2x_ are simulated. The main rock layers of the side slopes, including P_t_^21^ and Pt_21_^10^, are mainly of masses IV2, IV1 and III2. The computational model is divided into 753,839 units and 147,449 nodes. The layered rock masses are modeled with elastic–plastic lamina planes, which can simulate the damage behavior (shear and tension) along the lamina planes and the rock masses, and the anisotropic characteristics of the rock strength are considered. Figure 19a–d shows the horizontal sections of the numerically computed model at elevations 895 m, 865 m, 850 m, and 828 m, respectively.

**Table 1:** Rock mass parameters of various strata.

Rock layer	Unloading relaxation	Unit weight(kN/m^3^)	Modulus of deformation E(GPa)		Poisson's ratio *μ*	Shear strength			
						*f*'		*c*'(MPa)	
			Geological suggestion	Inversion parameter		Geological suggestion	Inversion parameter	Geological suggestion	Inversion parameter
Pt_21_^10^(IV2)	Non-unloading	26.8	1–2	2	0.33	0.5–0.7	0.7	0.2–0.4	0.4
	Unloading	26.6	/	1	0.35	/	0.42	/	0.2
Pt_21_^10^(IV1)	Non-unloading	26.8	3–5	5	0.3	0.7–0.8	0.7	0.4–0.7	0.7
	Unloading	26.7	/	2.5	0.33	/	0.42	/	0.35
Pt_21_^10^(III2)	Non-unloading	26.8	5–7	7	0.26	0.8–0.9	1	0.7–0.9	1
	Unloading	26.7	/	4	0.3	/	0.8	/	0.7
Pt_21_(IV2)	Non-unloading	26.8	1–2	2	0.33	0.5–0.7	0.7	0.2–0.4	0.4
	Unloading	26.6	/	1	0.35	/	0.42	/	0.2
Pt_21_(IV1)	Non-unloading	26.8	3–5	5	0.3	0.7–0.8	0.7	0.4–0.7	0.7
	Unloading	26.7	/	2.5	0.33	/	0.42	/	0.35
Pt_21_(III2)	Non-unloading	26.8	5–7	7	0.26	0.8–0.9	1	0.7–0.9	1
	Unloading	26.7	/	4	0.3	/	0.8	/	0.7
Z_2g_(IV2)	Non-unloading	26.8	1–2	2	0.33	0.5–0.7	0.7	0.2–0.4	0.4
	Unloading	26.6	/	1	0.35	/	0.42	/	0.2
Cataclastic rock–silty rock in Huashan 'gou fault	Non-unloading	26.6	1	1	0.33	0.5	0.5	0.2	0.2
	Unloading	26.6	/	0.6	0.35	/	0.25	/	0.1
Huashangou fault-mud mixed with cuttings		20	0.15	0.15	0.36	0.25–0.35	0.25	0.01–0.05	0.01
Z_2d_		27.3	5–7	10	0.3	0.8–0.9	1	0.7–0.9	1
P_3em_, P_2y_		27	14–18	18	0.25	1.0–1.2	1.2	1.2–1.4	1.4
J_2x_, J_1_, T_3bg_		27	5–10	10	0.28	0.8–1	1	0.5–0.7	1

In this paper, the stability coefficient calculated by strength reduction method is used as the evaluation standard for slope stability. Strength reduction method is a key step in slope stability calculation. When calculating slope stability coefficient in FALC^3D^, the initial stress state under the condition of self-weight is calculated first. Then, the parameters of cohesion (c) and internal friction Angle (φ) of rock and soil mass are gradually reduced by strength reduction method under this stress state until the slope is unstable, and the slope stability coefficient is obtained.

**Figure 20:**
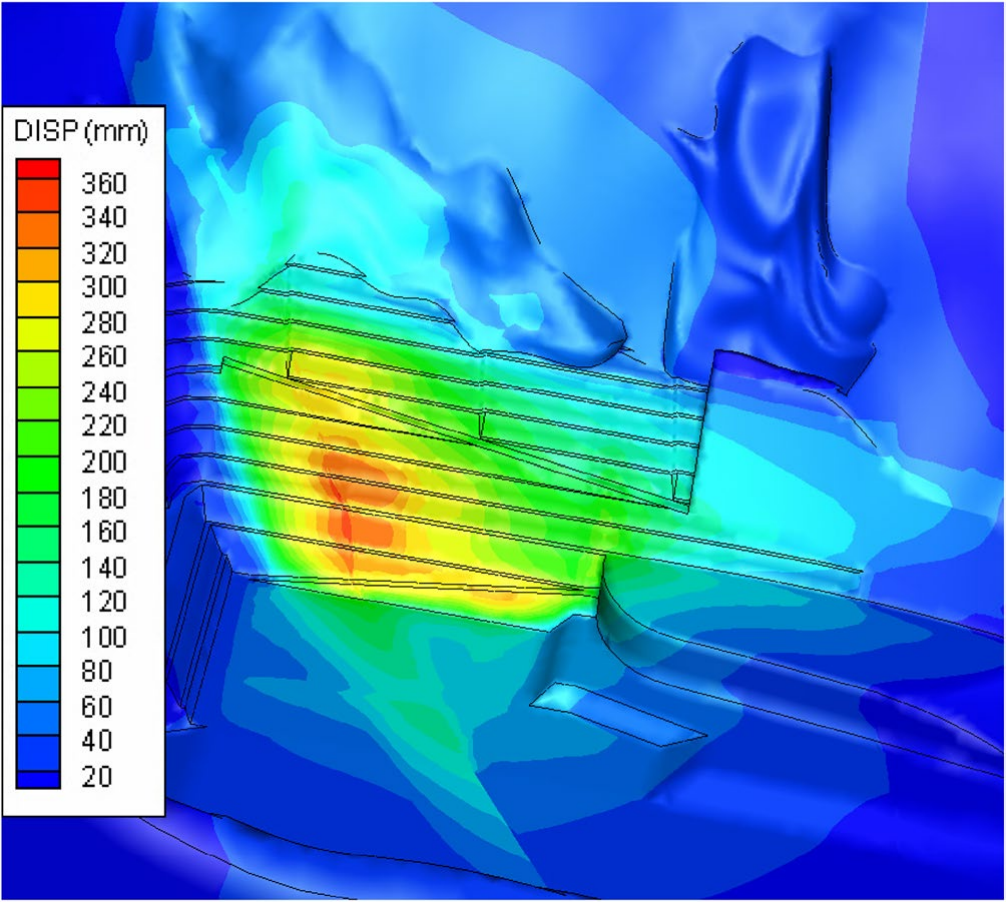
Total displacement cloud of slope.

The network cloud diagram of the overall total displacement of the slope is shown in Fig. 20. The depth of the plastic zone of the slope is 40–50 m, and the rock body of the slope shows a downward deformation trend toward the outside of the slope. When FLAC^3D^ software performs iterative calculation, the rock mass strength is continuously reduced until the slope is in an unstable state, and the calculation does not converge at this time, and the reduction coefficient at this time is the slope stability coefficient. In addition, the iterative reduction process of shear strength can be used to analyze the progressive failure process of slope. Combined with the characteristics of the finite difference procedure, according to the convergence criterion of the characteristic points and the displacement trend, it is determined that the safety coefficient of the slope is about 1.3, which meets the construction safety standard. During the construction process on site, the manual excavation causes the shallow surface of the slope to be affected to a certain extent and produces the unloading relaxation effect, and the shallow surface displacement is larger and shows a gradually decreasing trend. Because F6 is at the reverse slope, the bottom of the upper slope is "reverse" structure, and the slip force of the upper slope forms an obtuse angle with the direction of the bottom slip surface, which can only deform outward along the free side of the slope and continuously squeeze the fault and the lower slope rock body. When the performance of the lower rock body is better, the stability of the slope is also better. Combining with the geological conditions, monitoring data analysis and numerical simulation results, the deformation of the left bank slope has predominantly converged, and there is no structural surface of large scale such as fault that tends to be outward in the slope, so there is no overall stability problem of the slope. However, when the whole rock body under the foot is severely deformed or damaged due to unloading and relaxation, the upper slope has the possibility of continuous deformation.

#### Slope deformation analysis of the water cushion pond behind the dam

**Figure 21:**
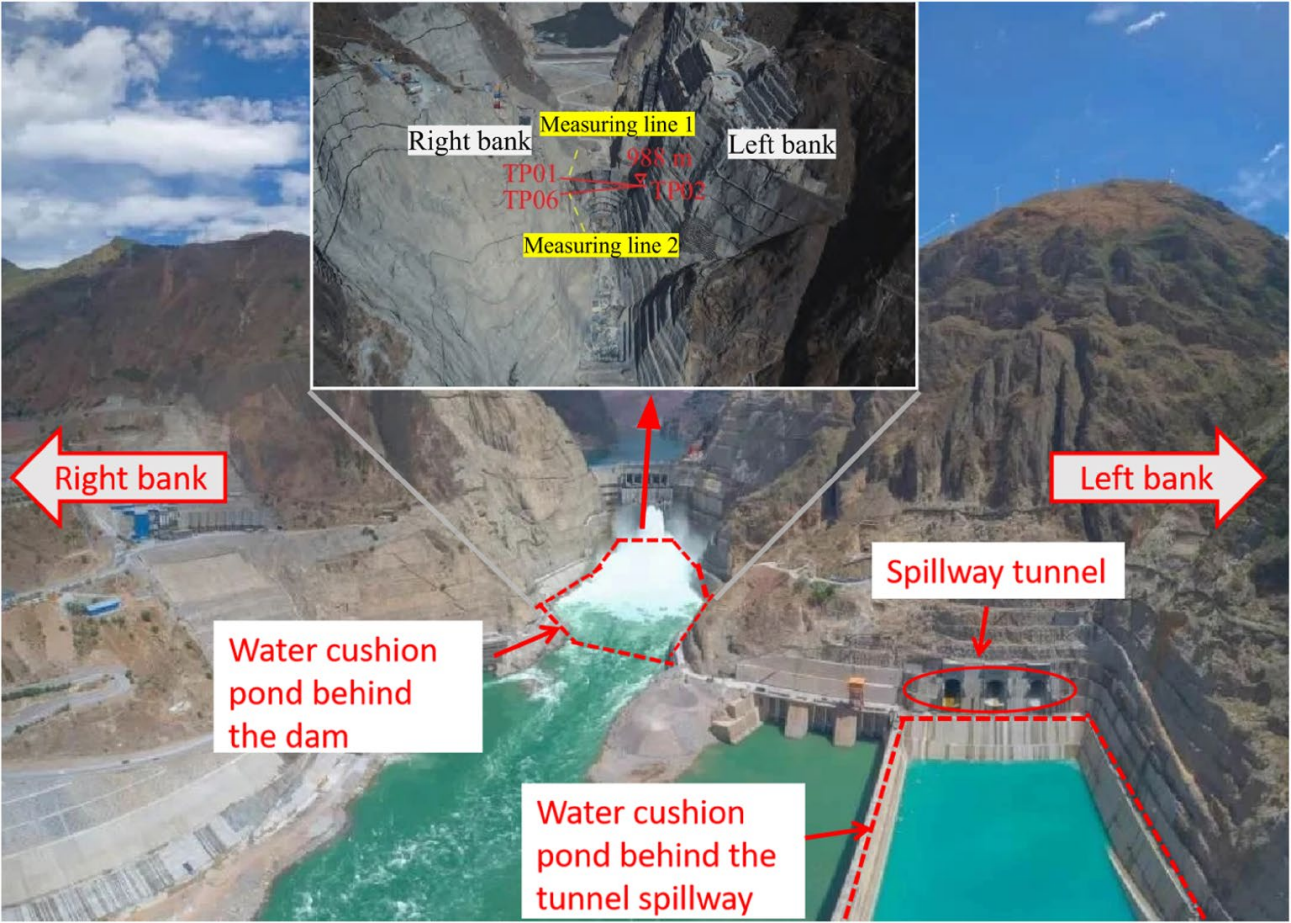
Location of water cushion ponds on the back side of the dam.

**Figure 22:**
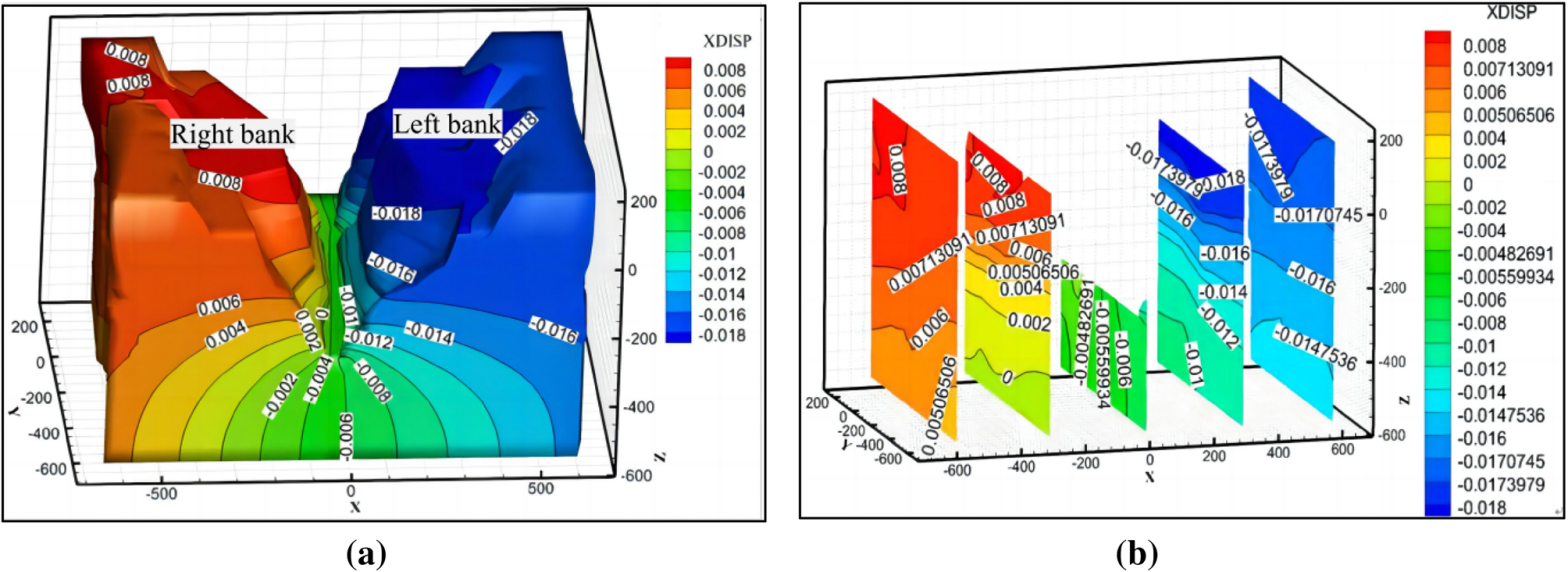
Numerical calculation model of water cushion pond on the back side of the dam: (**a**) Cloud image of model displacement in the X direction; (**b**) Sectional-plane cloud image of displacement contour line in the X direction of the model.

The horizontal spacing between the left and right sides of the hill of the water cushion pond at the outlet of the flood cave is too large, which is not conducive to numerical modeling, and it is difficult to accurately estimate the deformation and displacement of the slopes of the river valley on the left and right sides of the water cushion pond of the flood cave by only relying on the monitoring points. Meanwhile, the horizontal spacing between the side slopes on both sides of the water cushion pond at the back side of the dam is small, as shown in Fig. 21. The construction organization also arranged three sets of displacement monitoring points at the left and right ends of the vertical surface of the central course of Jinsha River. Combining the numerical simulation method with the field deformation monitoring, the deformation of the high side slopes on both sides of the water cushion pond behind the dam was compared and analyzed. The displacement diagram of the established numerical simulation model along the X direction is shown in Fig. 22a and b; the superposition of the absolute values of the slope deformation of the left bank and the right bank sloping toward the valley (i.e., the forward direction) is the valley deformation. In the numerical simulation of the water cushion pond behind the dam, the creep time is set through the creep command after the elastic–plastic calculation, and the creep time is referred to the real monitoring time on site. In this case, a total of 31 months between June 2016 and March 2019 were the actual monitoring time of the actual displacement on both sides, and 19,100 time steps were experienced in the elastic–plastic calculation using FLAC^3D^ software. At this time, the creep simulation calculation time is about 31 months, which can correspond to the actual deformation time of the water cushion pond slope behind the dam.

**Figure 23:**
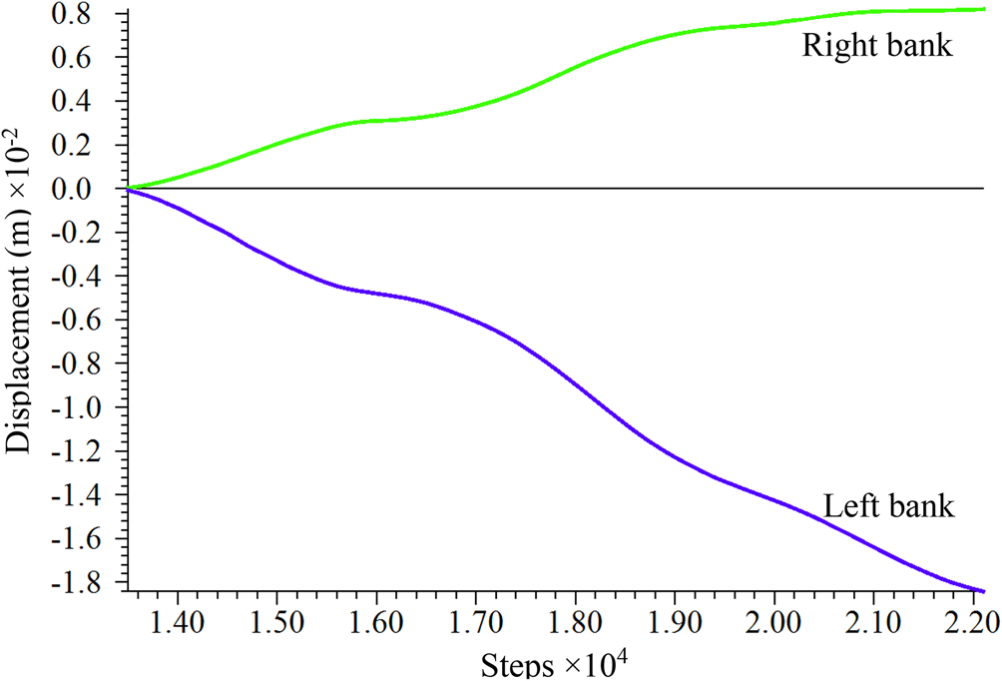
Horizontal displacement curve of 990 m elevation on the left and right bank.

**Figure 24:**
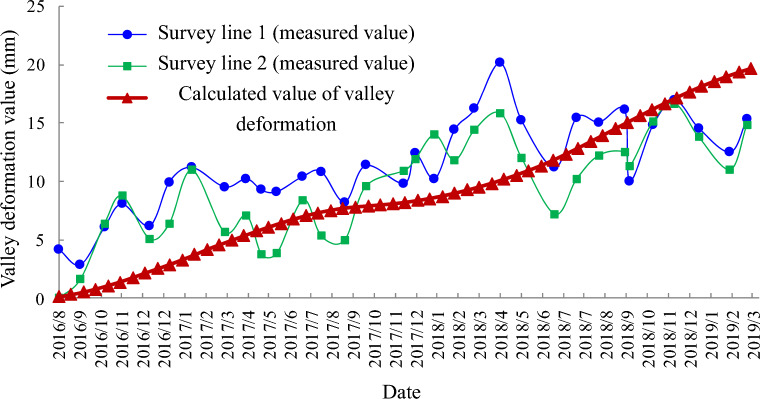
Comparison of field measurements and calculated valley deformation values (990 m post-dam water cushion pool elevation).

**Figure 25:**
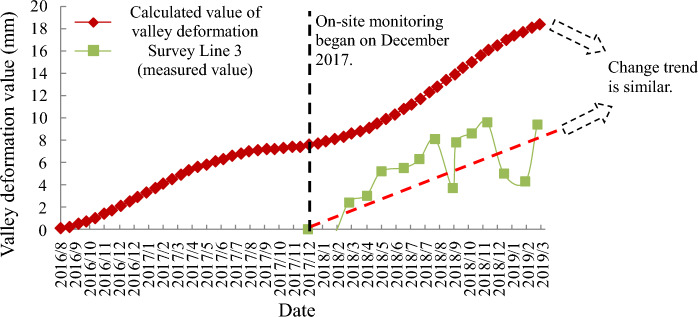
Comparison of field measured values and calculated values of valley deformation (post-dam water cushion pool elevation 900 m).

Comparing the calculated values of horizontal deformation of the left and right bank measurement points (990 m) obtained from numerical simulation, as shown in Fig. 23, the right bank deformed toward the valley with positive displacement values, and the left bank also deformed toward the valley with negative displacement values. The absolute values of the two deformations were superimposed toward the valley direction to obtain the change curve of the calculated values of valley deformation and compared with the measured values of the two measurement lines (measurement line 1 and measurement line 2) monitored by the water cushion pool behind the dam at the same elevation position, as shown in Fig. 24. Where the valley deformation, positive values are contraction and negative values are expansion. The comparison shows that there is a certain volatility in the field monitoring of valley deformation data; however, the overall rising trend of displacement of both measurement lines is consistent with the change of simulation results. The valley deformation, both numerically calculated and measured, did not exceed 20 mm. The absolute values at 900 m above sea level behind the dam were superimposed to obtain the variation curve of the calculated values of the valley and compared with the measured values of the valley measurement line 3 at the site elevation of 907 m, as shown in Fig. 25. Since the on-site monitoring of Line 3 started in December 2017, while the calculation time for the set of calculated values of valley deformation started in September 2016, a comparative analysis of the same period is not possible, but it is possible to compare the trends of both. Since December 2017, the calculated values have similar trends to the actual measured values in the field. Comparatively accurate comparison of the valleyward shrinkage deformation at 990 m and 900 m elevation shows that the FLAC^3D^ simulation results and the actual engineering situation can be well corroborated with each other.

**Figure 26:**
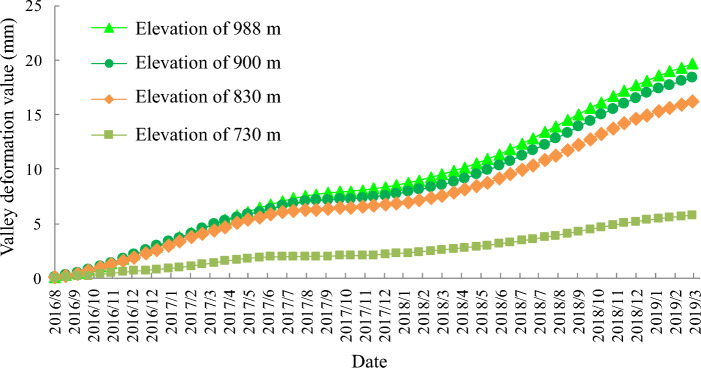
Calculated values of valley deformation at different elevations.

By superimposing the calculated values of valley deformation on both sides of the reservoir behind the dam at different elevations (upper 990 m, upper 900 m, lower 830 m, lower 730 m), the valley deformation curves at each elevation can be obtained as shown in Fig. 26. The valley shrinkage pattern of each elevation is similar, showing a slow increasing trend. 830 m and above height valley shrinkage deformation is not much different, but the difference with 730 m height valley shrinkage deformation is obvious. The valley deformation at altitude 990 m is the largest, close to 20 mm, and the valley contraction at the bottom 730 m is the smallest, only 5 mm.

## Deformation evolution mechanism

### Water seepage effect

**Figure 27:**
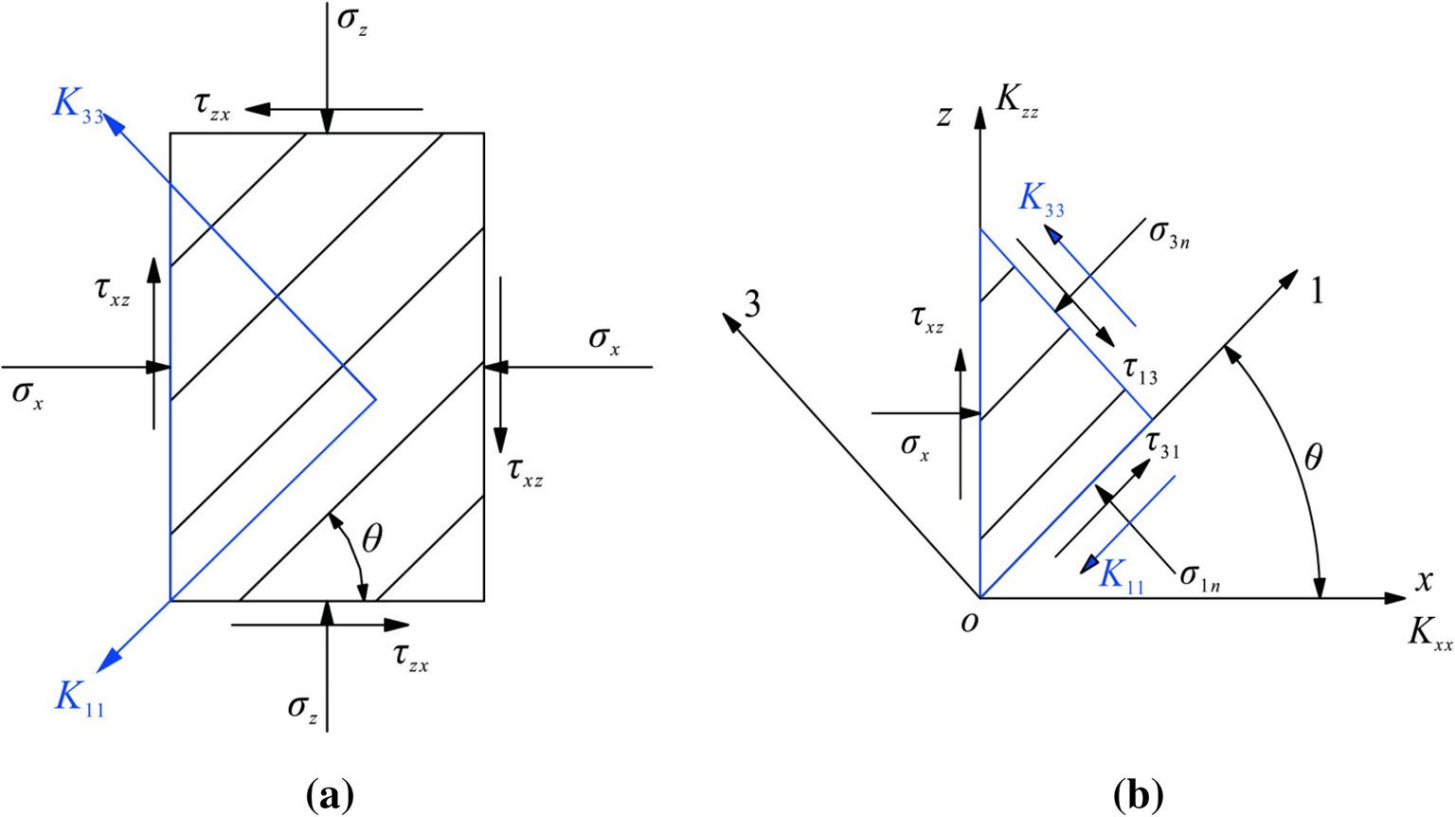
Equivalent continuous medium mechanical model of a typical layered rock mass: (**a**) Stress state of layered rock. σ_x_ is horizontal positive stress; σ_z_ is vertical positive stress; τ_zx_, zx-directional shear stress; τ_xz_ indicates xz-directional shear stress; θ is the dip angle of the layered structure face; K_11_ and K_33_ are equivalent permeability coefficients along and perpendicular to the direction of the layered structure face, respectively; (**b**) Equilibrium state of layered rock unit. σ_1n_ is normal shear stress; σ_3n_ is horizontal tangential stress; τ_31_ indicates tangential shear stress; τ_13_ indicates normal shear stress; K_xx_ is horizontal permeability factor and K_zz_ is vertical permeability factor. xOz is the overall coordinate system and 1O3 is the local coordinate system.

Seepage is an essential factor affecting the stability of layered slope. Especially for the slope on both sides of the dam reservoir area, the seepage of water will significantly affect the stability and displacement of the slope. In this case, the shrinkage deformation value of the valley increased significantly after the cushion pond began to store water on June 24, 2017, which well confirms this rule. On the one hand, the change of the stress state of the rock body causes deformation of the rock body and leads to the change of the geometric parameters of the fissure, especially the opening, which changes the permeability of the rock body and eventually leads to the change of the seepage field of the rock body; on the other hand, the permeable water pressure generated by the seepage of groundwater in the bond surface and fissure can change the initial stress state of the rock body, even due to the difference between different soil or rock layers. The variability of permeability coefficients makes it possible for water to form seepage channels parallel to the slope at the interface of soil or rock layers^47^. Due to the seepage control characteristics of fissures, layered rock masses exhibit strong permeability anisotropy in the direction along the laminae and in the direction of the vertical laminae. Based on the classification of rock mass in the study area, the layered rock mass is simplified to the equivalent continuum mechanical model. Figure 27a shows the stress state of typical stratified rock mass, and Fig. 27b shows the equilibrium state of stratified rock mass elements.

The fractured rock masses play a dominant role in seepage by the stress in the direction perpendicular to the fracture surface under the action of three-way stress, so the sudden change in the stress field of the layered geological body also causes the change in the seepage field of fractured water^40,48^. In this case, the excavation of the slope of the water cushion pool of the flood cave releases the strain energy of the geotechnical body, which causes the opening and misalignment of the original structural surface within the rock body, as well as the expansion of primary joint fractures or fresh fractures, thus triggering non-uniform seepage and bringing negative impacts to the layered slope. The coupling effect of engineering disturbance and water seepage has a negative effect on the bottom stability of the slope, and the specific performance in this case is the increase of displacement of the valley deformation of the mountain body above the elevation of 1070 m on the left bank after the pool storage.

### Excavation disturbance

**Figure 28:**
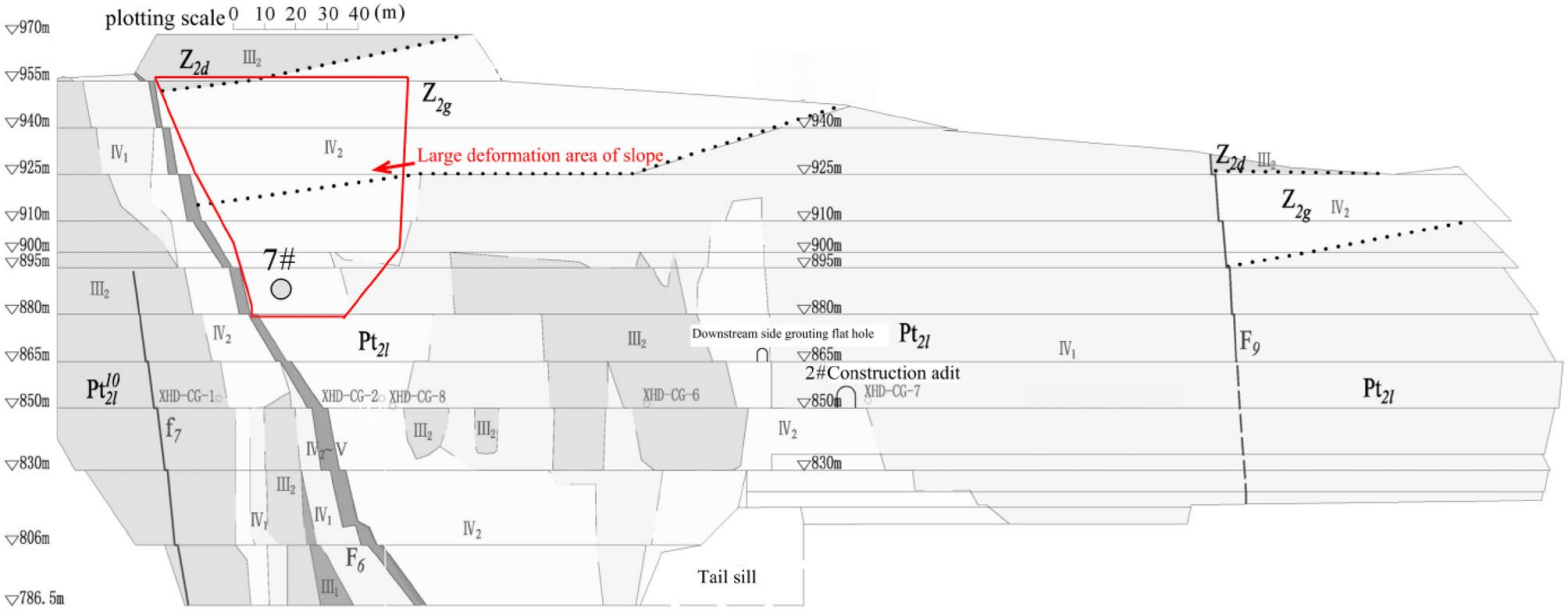
Rock quality of the left side slope of the water pad pond of the spillway tunnel^34^.

**Figure 29:**
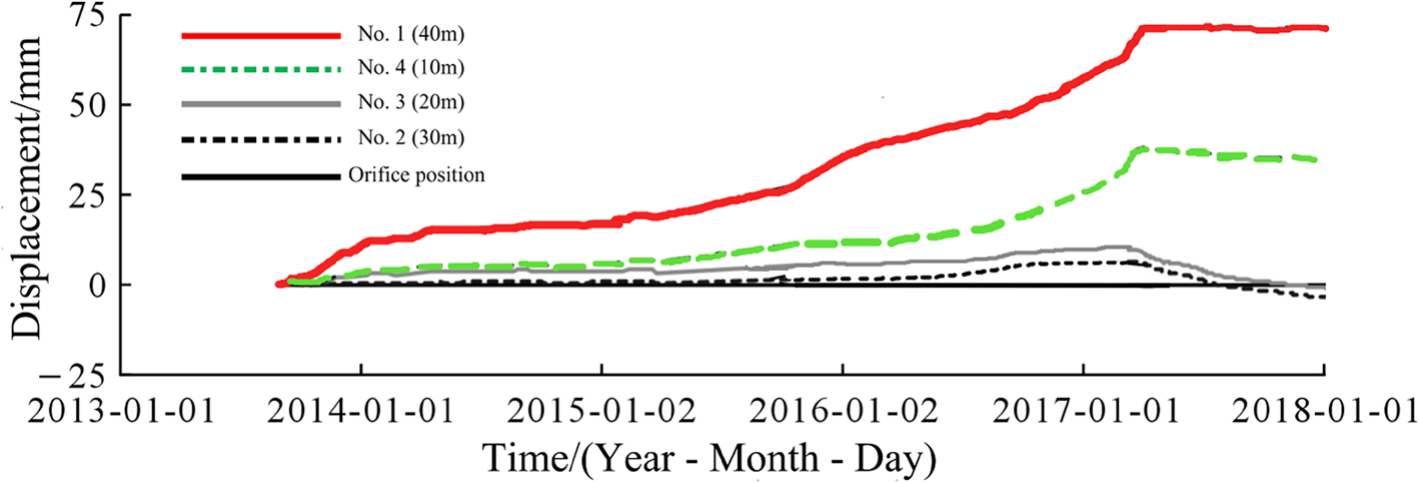
Rock deformation measured by multi-point displacement meter in borehole No. 7^34^.

With reference to the preliminary detailed geological investigation and related literature^34,35^, it can be determined that the construction operation behaviors such as excavation and blasting are more disturbing to the slope. The rock body on the downstream side of the F6 fault on the left side of the spillway tunnel water pad pond is mainly type IV2 with poor quality, and this type of rock body accounts for 32.6% of the whole slope (see Fig. 28). The parts of the slope with larger deformation are marked with red lines (corresponding to the red circle part of Fig. 13). The area with larger deformation on the slope surface has higher consistency with the area with larger deformation of the rock mass, and the larger deformation in this area is related to the F6 fault, which is mainly generated by the unloading relaxation of the rock mass caused by the excavation in this area. At an elevation of 891.6 m on the downstream side immediately adjacent to the F6 fault, physical exploration was conducted using anchor holes (hole numbered 7 in Fig. 28), and the relaxation depth was found to be about 16.4 m. The acoustic wave velocity in the relaxation zone was 2600–3200 m/s, and the acoustic wave velocity in the non-relaxation zone was 3100–5000 m/s. In order to verify the relaxation depth of rock unloading relaxation caused by excavation, a multi-point displacement meter was set in physical exploration hole number 7 In order to verify the relaxation depth of rock unloading caused by excavation, the rock deformation measured by setting multi-point displacement meter in hole 7 of physical exploration is shown in Fig. 29, and the distance marked in the figure refers to the distance from each monitoring point of displacement meter to the deep anchorage head. It can be seen that the maximum deformation after the excavation is completed is about 73 mm. 16.4 m is the depth of loosening and unloading determined in the exploration hole No. 7, which confirms the existence of rock unloading phenomenon in this area and determines that the depth of loosening and unloading is within the range of 0–20 m of rock deformation. Due to the unloading relaxation caused by excavation and other factors, as well as the poor quality of the rocks in this area downstream of the F6 fracture, large tensile deformation and surface deformation occurred in the rocks near section 4–4 of the first phase downstream of the F6 fracture.

**Figure 30:**
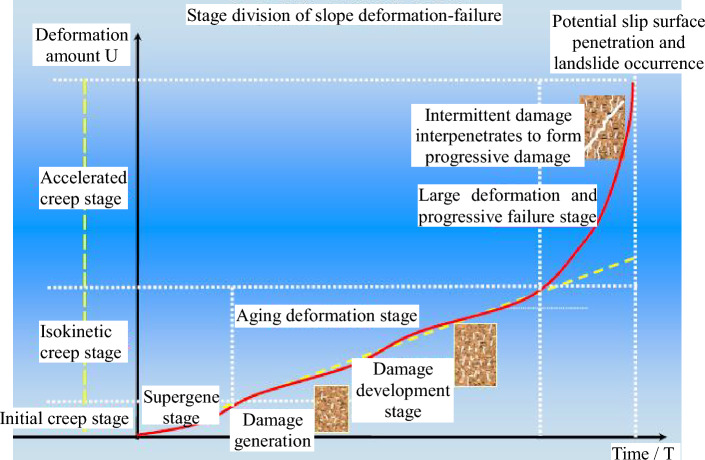
Evolution of deformation-destruction of layered slopes.

The deformation evolution of the slope is a common process of microscopic damage and fracture development of the rock mass, continuous decrease of macroscopic mechanical strength, and non-homogeneous infiltration of fracture water. The overall evolution of long-term deformation and damage of the slope is plotted according to the trend of the displacement data of each monitoring point, as shown in Fig. 30. Due to the gradual change of rock slope with time, the sliding surface of such slope gradually forms after a long time of incubation. For slopes with gradual evolution and deformation caused by external artificial excavation, if there is no obvious control structural surface within the slope, the key to influence the stability of the slope is to control the development of deformation. When the slope is stable, it will stay in the gestation stage or development stage of damage development, and it is not easy to further develop to the final stage of large-scale, progressive and large deformation, similar to the change of multi-point displacement meter data in Probe Hole 7 (see Fig. 29), which will converge when it grows to a certain value. If the deformation remains uncontrollable after the release of strain energy, the displacement will develop freely and the sliding surface will gradually multiply to completion, i.e., it will enter the irreversible state, thus the slope will lead to the overall damage. At present, the whole of Wudongde hand-excavated slope is in the stage of convergence of deformation, and no coherent and unified sliding surface is formed inside.

### Treatment measures

Due to the harsh geological conditions in the area where Wudongde Hydropower Station is located, geological hazards are frequent and easy to occur, which poses a great threat to safe construction and operation. Referring to the displacement of other layered slope projects^14,49^, the overall value of valley deformation in this project is not large and falls within the normal range, indicating that the treatment measures have achieved excellent results in controlling slope displacement.

As a large-scale high and steep slope, Wudongde dam must rely on management measures to maintain or solve the overall stability problem. According to the engineering practice experience and the deformation mechanism of laminated geotechnical slope, according to the idea of combining short-term emergency reinforcement and long-term comprehensive management, the management measures are taken:. The main principles adopted in the design are: (1) Following the principle of water treatment before slope treatment, it is necessary to minimise the infiltration of surface water into the rock body of the slope on the one hand, and exclude the groundwater in the rock body of the slope in time on the other hand. (2) For the unstable or poorly stable random blocks, positioning blocks and semi-positioned fast bodies exposed on the slope surface, anchor support or concrete spraying support is mainly adopted to reinforce them. (3) For the slope with a large range of tensile and shear stress area and plastic damaged area due to excavation and unloading, in order to improve the stress state, limit the tensile stress area and plastic area tracking cracks to form tensile cracks and further deteriorate the slope stability conditions, take pre-stressing anchors to be supportive treatment. (4) Fully consider the terrain, geological conditions, according to the requirements of the hub building structure arrangement and the results of the calculation and analysis of the excavation slope, the outlet slope of the spillway hole is scientifically and reasonably divided into sections, graded excavation support, in order to solve the overall self-stabilisation of high slope, to ensure that the construction period and the operation of the period of safety (Supplementary information).

**Figure 31:**
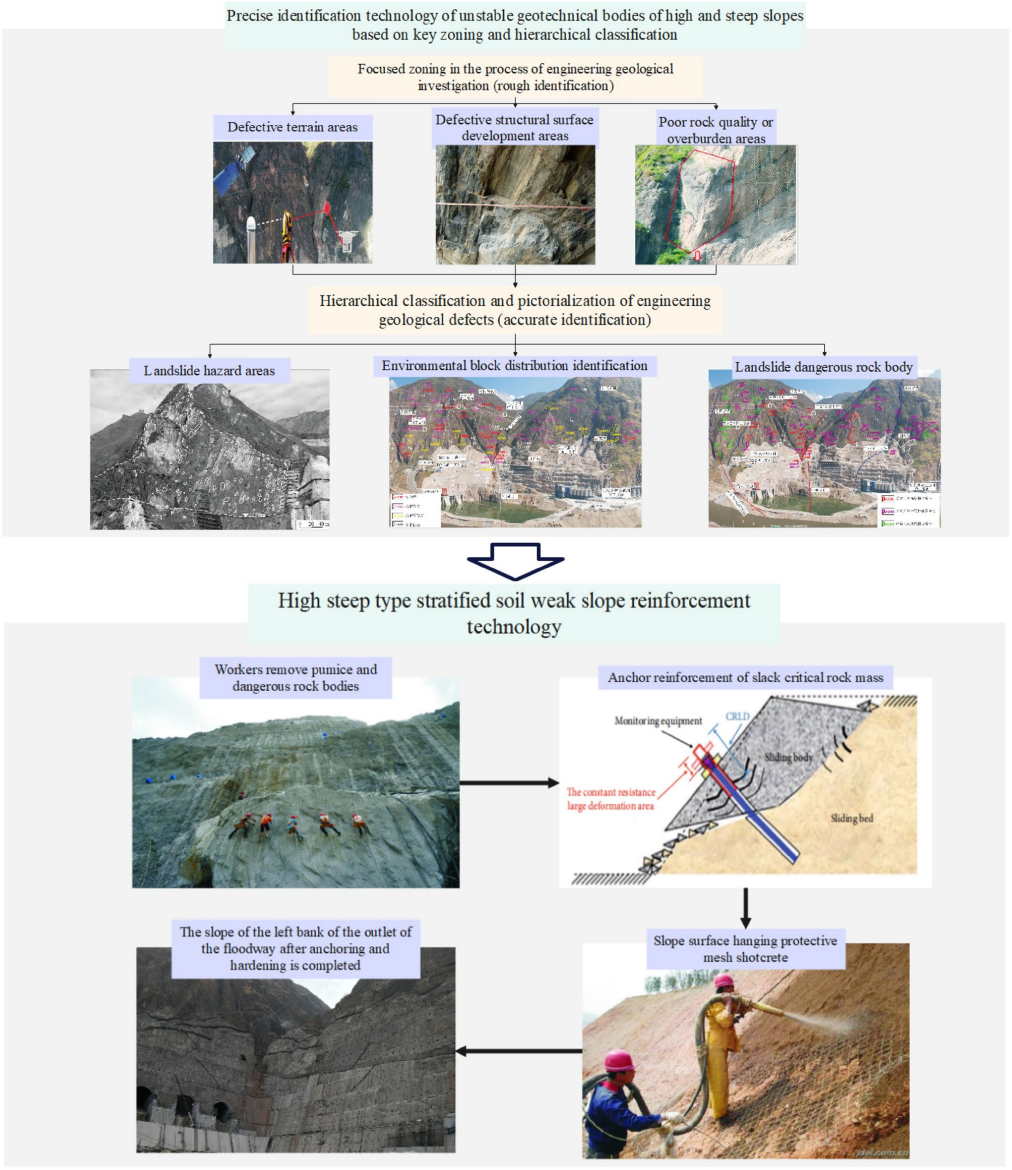
Precise identification and reinforcement technology for landslide hazard areas.

The construction organization has achieved positive results after implementing a series of treatment measures on site. The treatment measures mainly consist of two parts, which are precise identification technology for unstable geotechnical bodies and defective areas, and reinforcement technology such as anchoring of unstable rock bodies and slope hardening, as shown in Fig. 31. As of July 2019, the displacements of each monitoring point of the slopes on both sides of the reservoir area are at the convergence stage, and no landslides and crumbling disasters have occurred throughout the construction. All units of Wudongde Hydropower Station have been put into operation in 2021, and the monitoring indexes of the slopes in extra-high and steep environments are operating regularly.

## Conclusions

In this paper, the evolution mechanism of the stability and large deformation of high steep layered slopes is analyzed based on the measured displacement data of different rock bodies in the flood relief tunnel of Wudongde Hydropower Station, and the main conclusions are as follows.

1. From 2013 to 2019, the displacement of the natural mountain body (in the area with an elevation of more than 1070 m) changes smoothly in two directions (horizontal and vertical), and the deformation of the left bank towards the river valley is larger than that of the right bank during the same period, while there is no obvious sudden shift on the right bank. The natural mountains on both sides of the outlet of the spillway did not show any deformation greater than 50 mm during the whole construction process, indicating that the excavation and blasting construction of Wudongde dam has limited influence on the surrounding mountains. The slope in the manually excavated area on the left bank of the water cushion pond with an elevation of 1070 m or less are more susceptible to blasting, excavation and other engineering activities, and the deformation of the rock body and the surface deformation of the slope are more obvious. The deformation of the manually excavated area is clearly larger than that of the natural slope, and the maximum displacement of the multi-point displacement meter for measuring the deformation of the rock body occurs at the measuring point M11, with the maximum displacement of about 92.15 mm, and the maximum displacement of the surface monitoring pier for measuring the deformation of the slope surface occurs at the measuring point TP11, with the maximum displacement of about 312.5 mm, and the largest deformation is close to the deformation monitoring cross section of 4–4, i.e., the projecting ridge-like terrain, especially near the top of the opening line there, with the next highest amount of deformation in the mid-slope area. The deformation of the natural mountain body increased significantly after the filling of the water cushion pond; however, both the horizontal and vertical values were kept within 20 mm, and the risk of valley contraction deformation was within the overall controllable safety range.
2. By mean of finite element software, the stability of the left slope at the exit of the spillway tunnel is analyzed, and the safety factor of the slope is about 1.3. The absolute value of the displacement of both banks of the water cushion pond behind the dam to the valley direction, that is, the valley deformation, calculated by superimposed numerical simulation, can be seen that the valley deformation behind the dam increases slowly and shows a shrinking trend in general, with the maximum shrinking amount approaching 20 mm and located at an altitude of 990 m. Considering the large nonlinear deformation of rock mass, the valley deformation of water cushion pond behind dam is simulated by finite difference numerical analysis method, and the internal spatial evolution effect of deformation is shown. The calculated value of the valley deformation of the water cushion pond behind the dam is close to the field measured value.
3. Regarding the stability of layered rock slope, groundwater seepage is a highly significant factor, but the numerical simulation model involved in this paper does not consider the calculation of groundwater and the coupling of soil and water, and the scientific problems of layered rock slope need to be more studied. There is an obvious phenomenon of stratification and block in the slope studied in this paper. Now that Wudongde Hydropower Station has been completed and put into operation, it is suggested to strengthen the tracking investigation of water impoundment in the later stage and expand the scope of deformation monitoring in the dam area and reservoir area. After impoundment, the rock mass of layered rock and soil slope may be infiltrated and softened due to seepage, which needs to be further studied compared with other water conservancy projects.

### Supplementary Information

Supplementary Information.

## Data Availability

Data will be made available on request. Data supporting the results of this study can be obtained by contacting the frst author, Chen Ding. When sending an e-mail inquiry, be sure to state a valid reason and be brief and concise.
