# Supplementary figures and images for: Case study on long-term deformation monitoring and numerical simulation of layered rock slopes on both sides of Wudongde dam reservoir area
Source: Sci Rep. 2024 Mar 22;14:6909. doi: 10.1038/s41598-024-57598-7 (PMC10960035; doi:10.1038/s41598-024-57598-7)

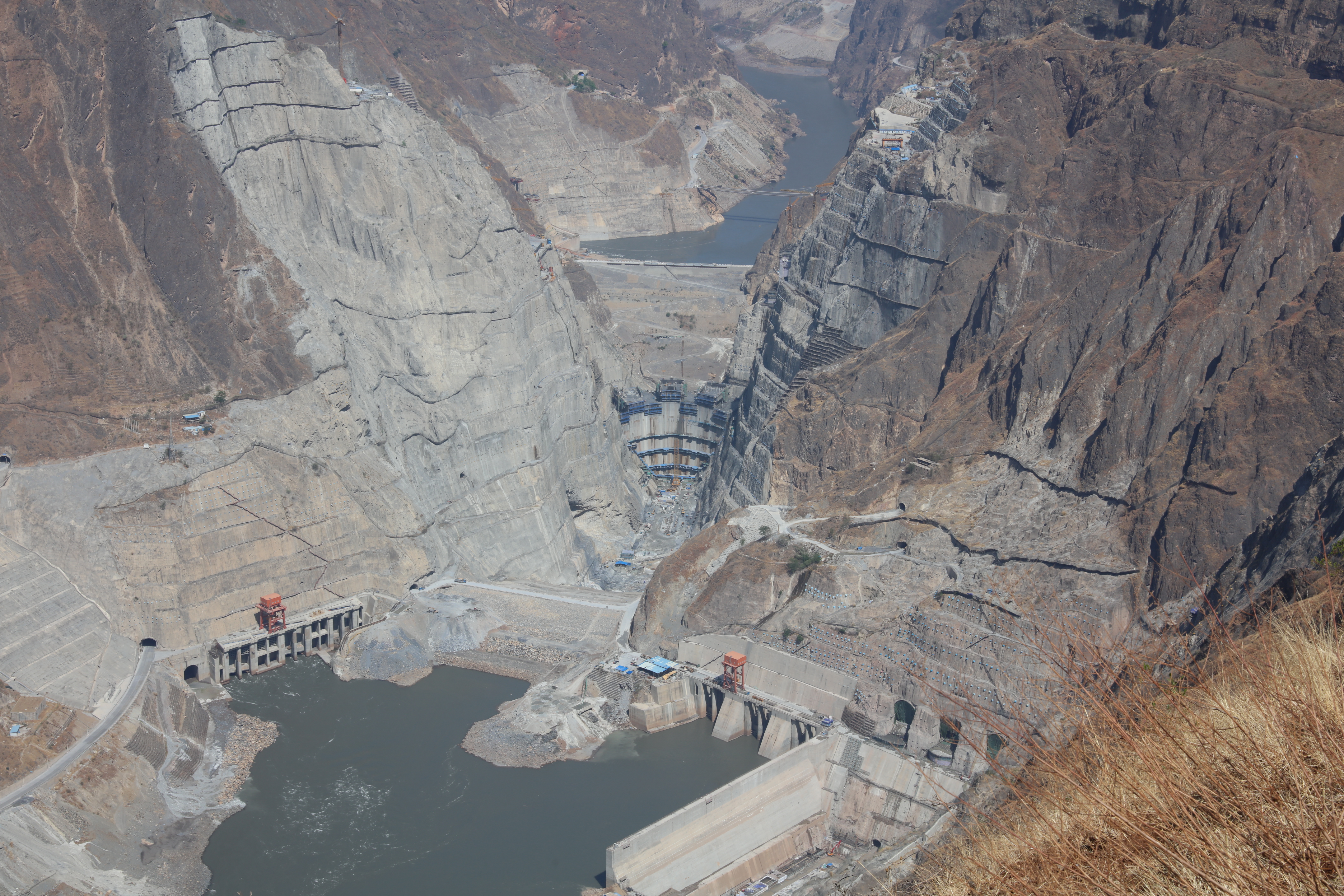

Supplement: Supplementary file 1 — Supplementary Information. [file 41598_2024_57598_MOESM1_ESM.zip › 6P5A6600.JPG]

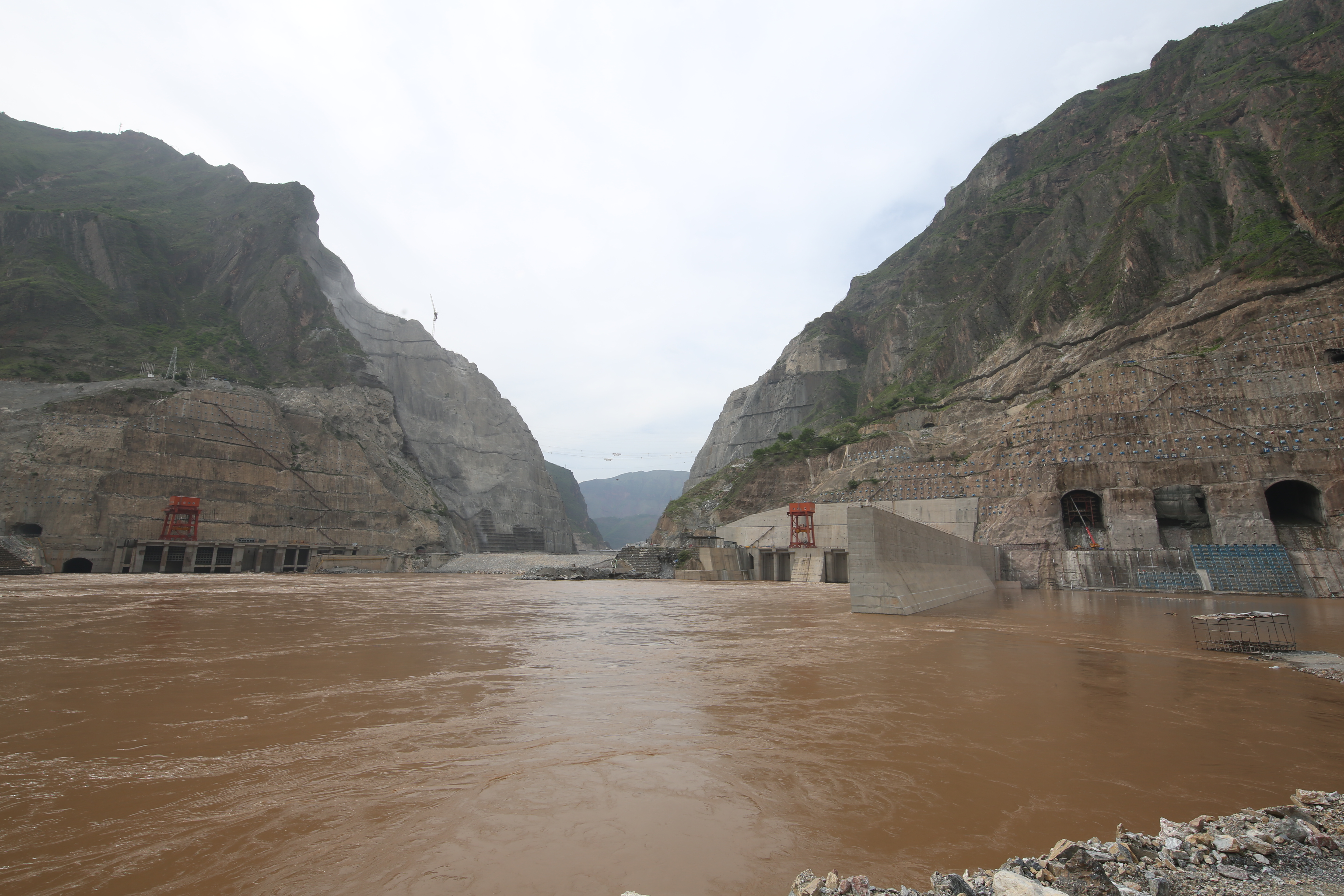

Supplement: Supplementary file 1 — Supplementary Information. [file 41598_2024_57598_MOESM1_ESM.zip › AL0A6048.JPG]

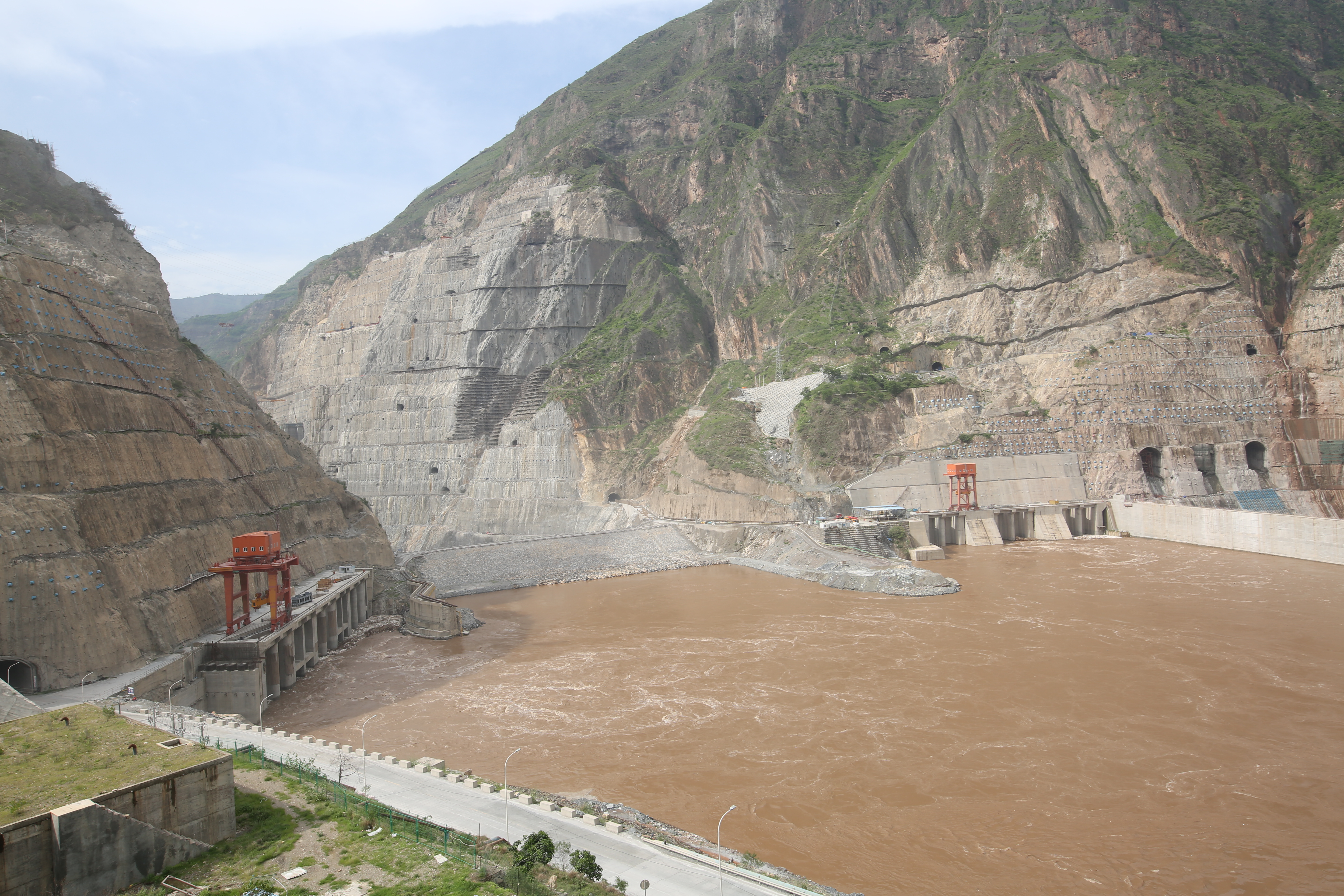

Supplement: Supplementary file 1 — Supplementary Information. [file 41598_2024_57598_MOESM1_ESM.zip › AL0A6057.JPG]

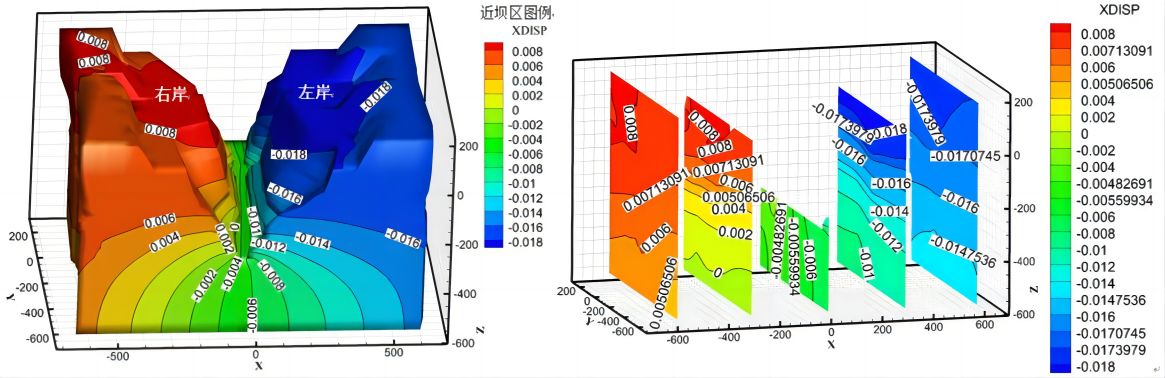

Supplement: Supplementary file 1 — Supplementary Information. [file 41598_2024_57598_MOESM1_ESM.zip › WPS拼图0.png]

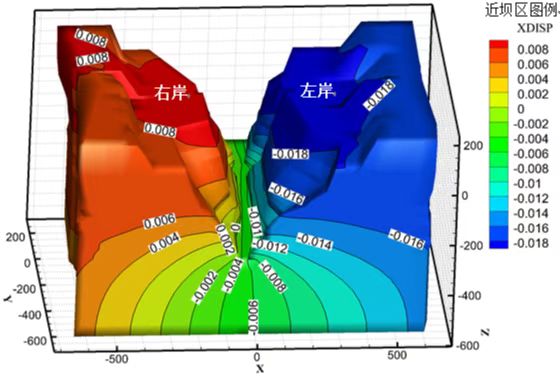

Supplement: Supplementary file 1 — Supplementary Information. [file 41598_2024_57598_MOESM1_ESM.zip › mmexport1665666480013.jpg]

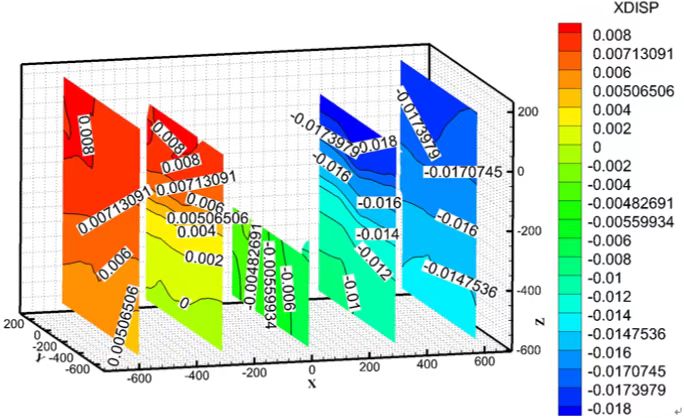

Supplement: Supplementary file 1 — Supplementary Information. [file 41598_2024_57598_MOESM1_ESM.zip › mmexport1665666483058.jpg]

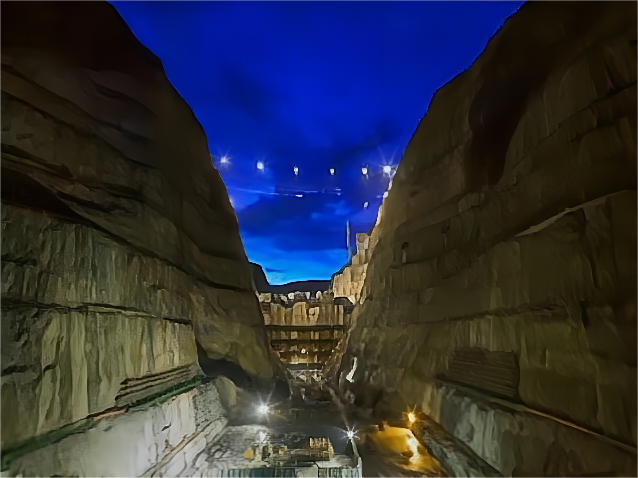

Supplement: Supplementary file 1 — Supplementary Information. [file 41598_2024_57598_MOESM1_ESM.zip › mmexport1665666486538(1).png]

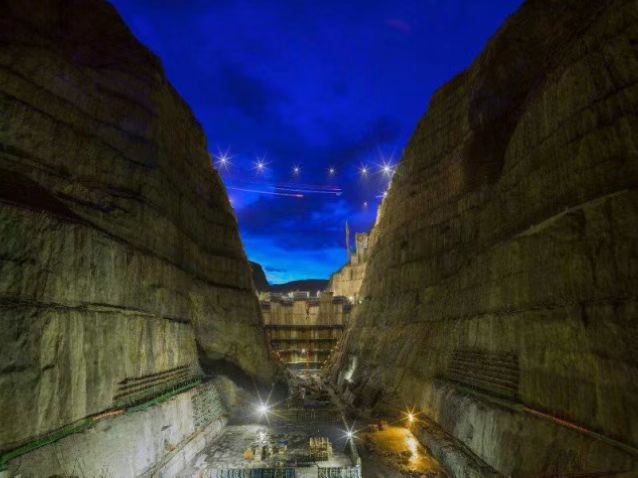

Supplement: Supplementary file 1 — Supplementary Information. [file 41598_2024_57598_MOESM1_ESM.zip › mmexport1665666486538.jpg]

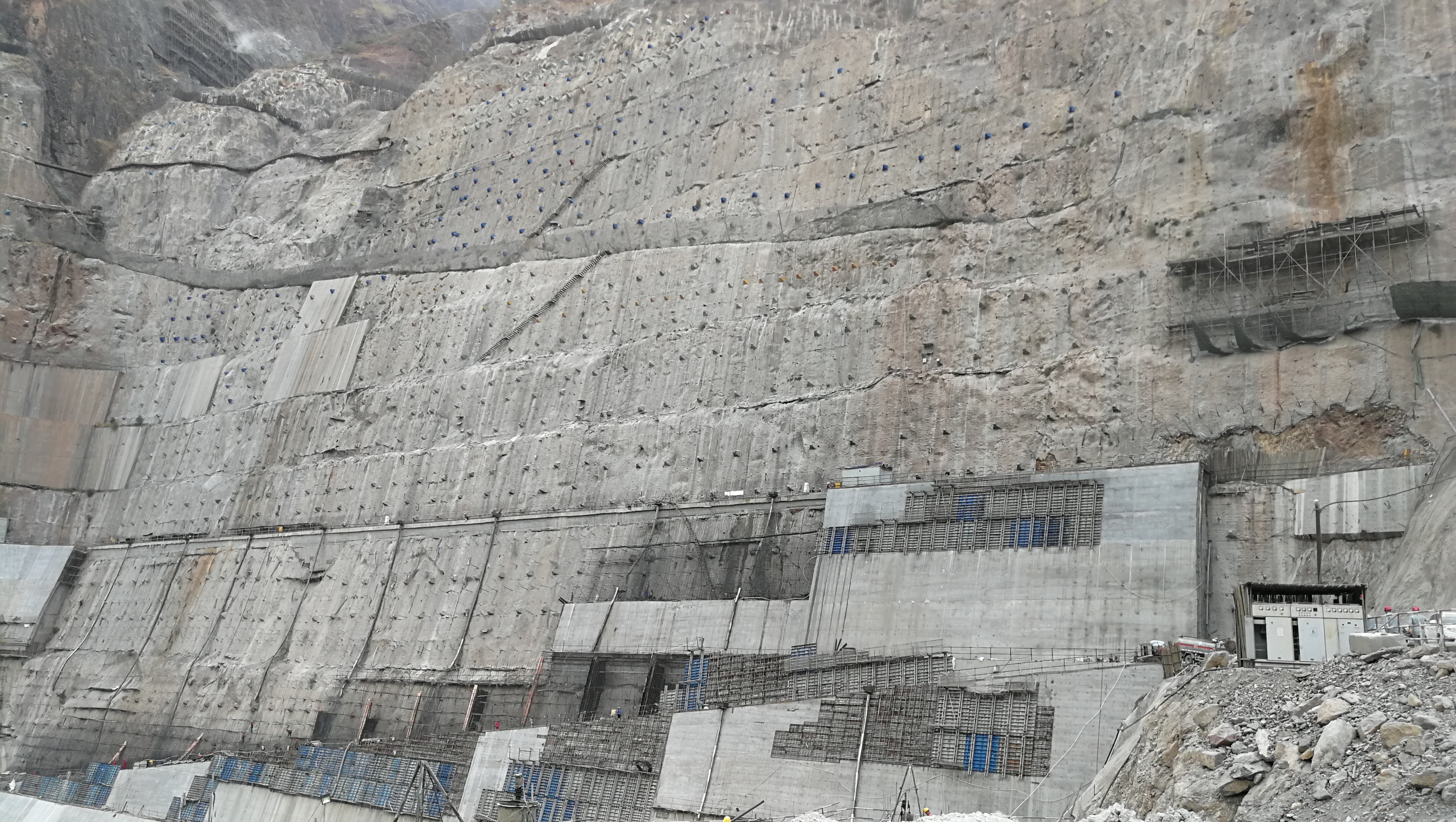

Supplement: Supplementary file 1 — Supplementary Information. [file 41598_2024_57598_MOESM1_ESM.zip › 泄洪洞出口侧面坡03.jpg]

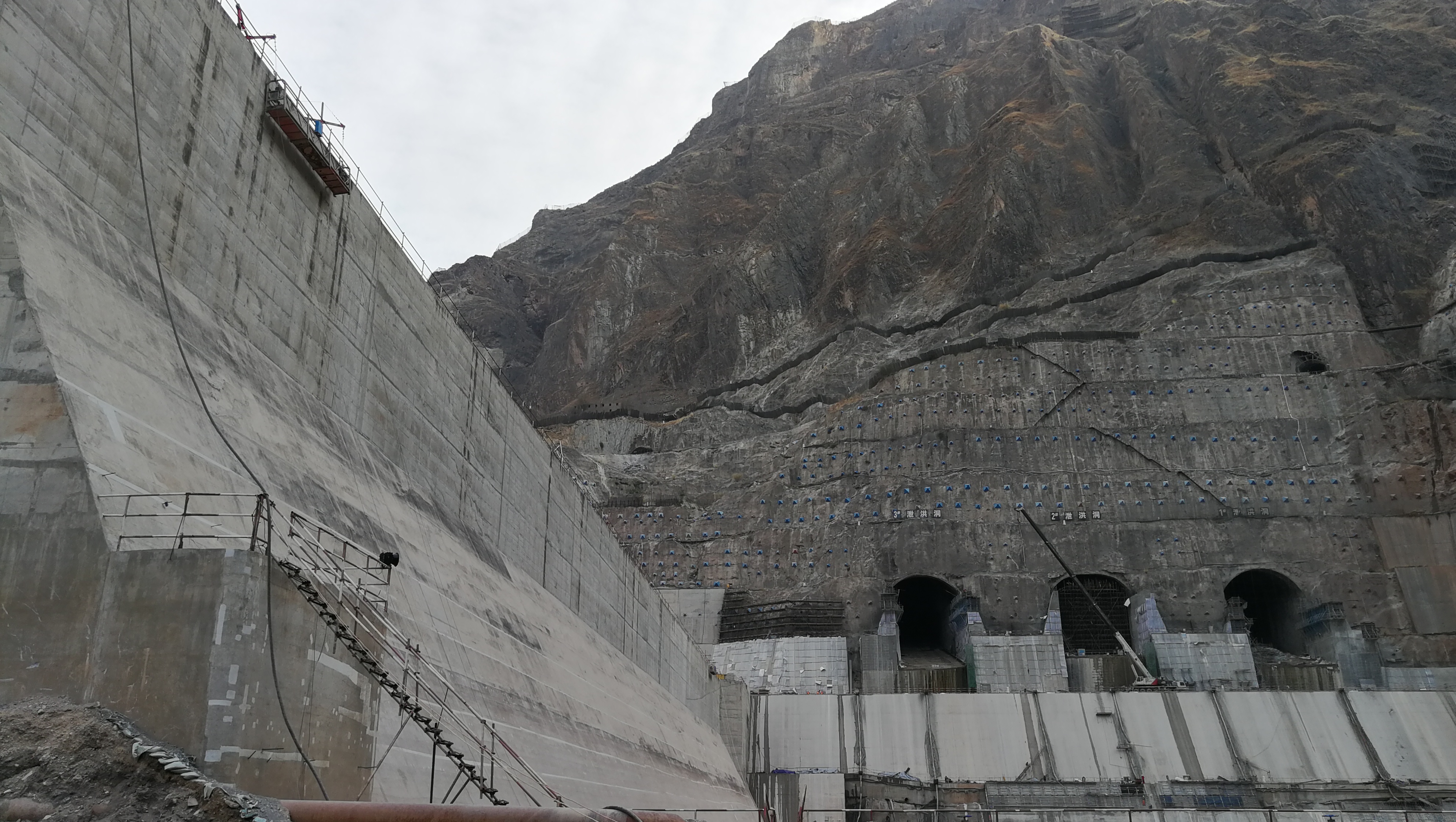

Supplement: Supplementary file 1 — Supplementary Information. [file 41598_2024_57598_MOESM1_ESM.zip › 泄洪洞出口正面02.jpg]

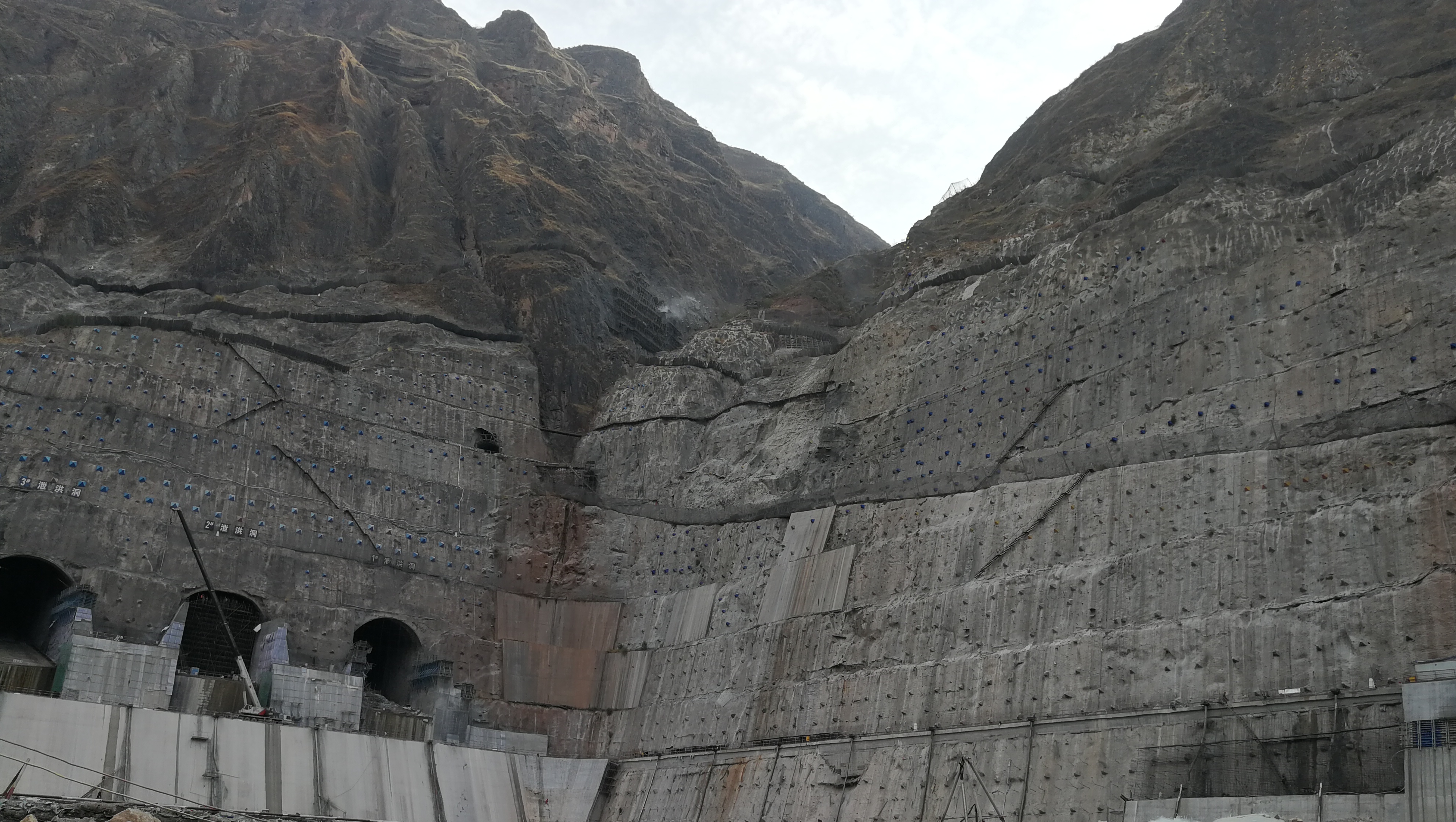

Supplement: Supplementary file 1 — Supplementary Information. [file 41598_2024_57598_MOESM1_ESM.zip › 泄洪洞出口正面03.jpg]

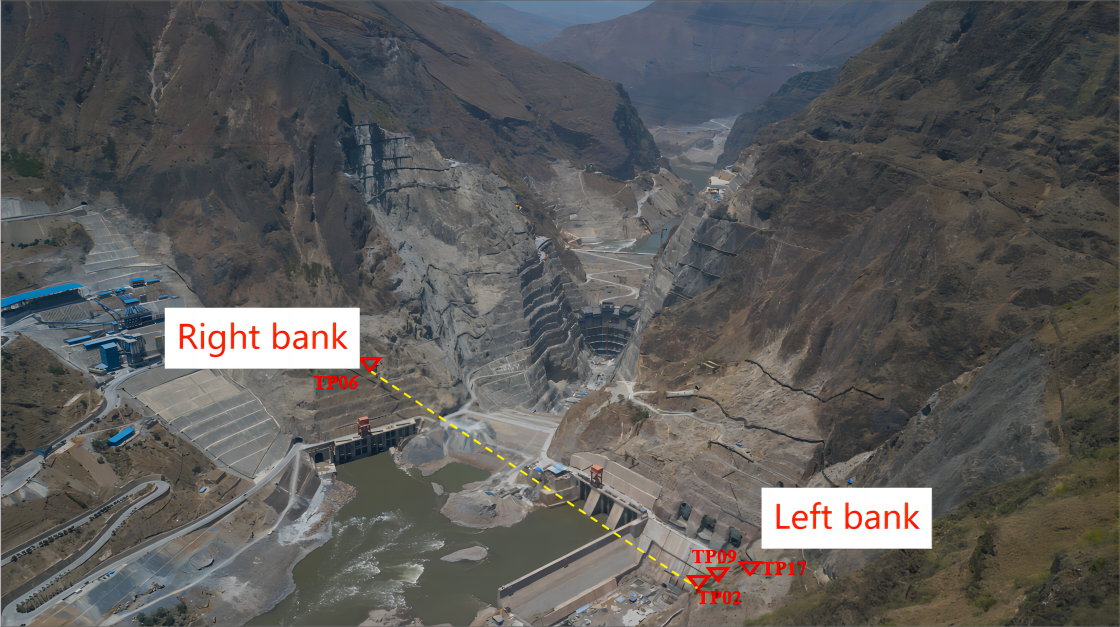

Supplement: Supplementary file 1 — Supplementary Information. [file 41598_2024_57598_MOESM1_ESM.zip › 补充图01(1).png]

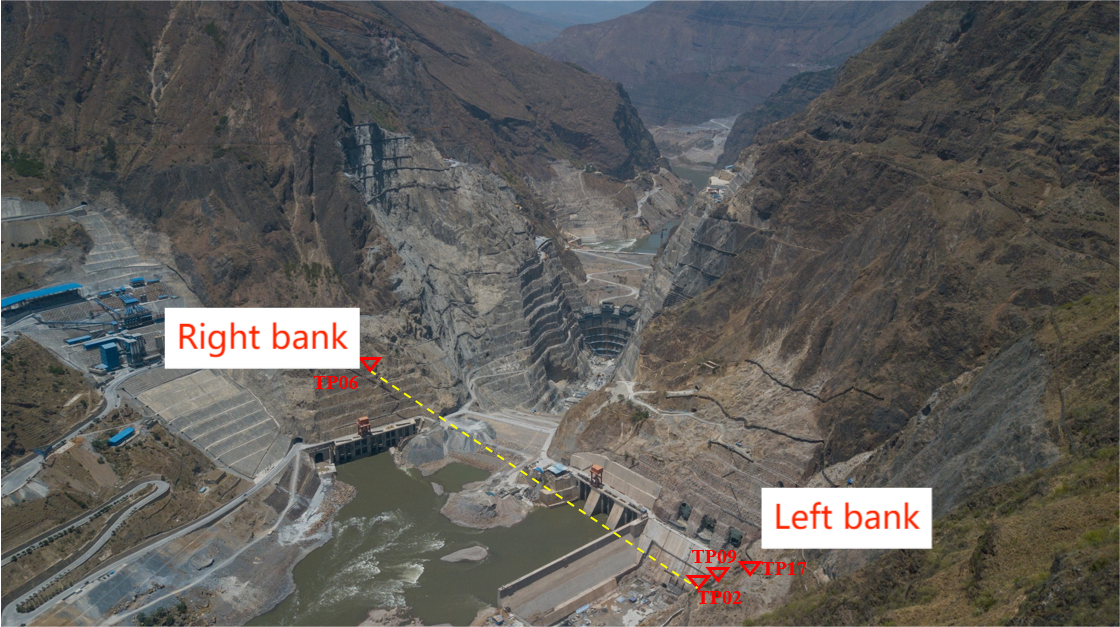

Supplement: Supplementary file 1 — Supplementary Information. [file 41598_2024_57598_MOESM1_ESM.zip › 补充图01.png]

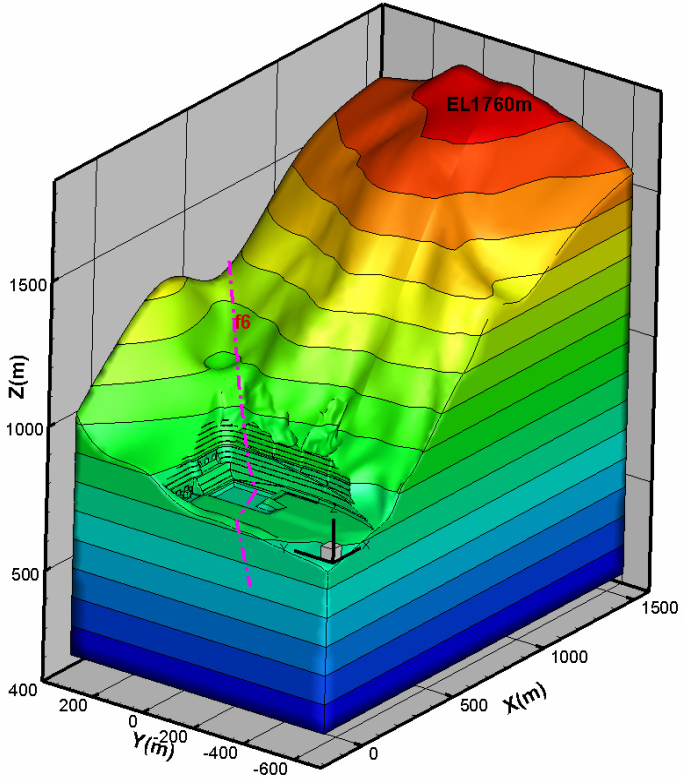

Supplement: Supplementary file 1 — Supplementary Information. [file 41598_2024_57598_MOESM1_ESM.zip › 补充图02.png]

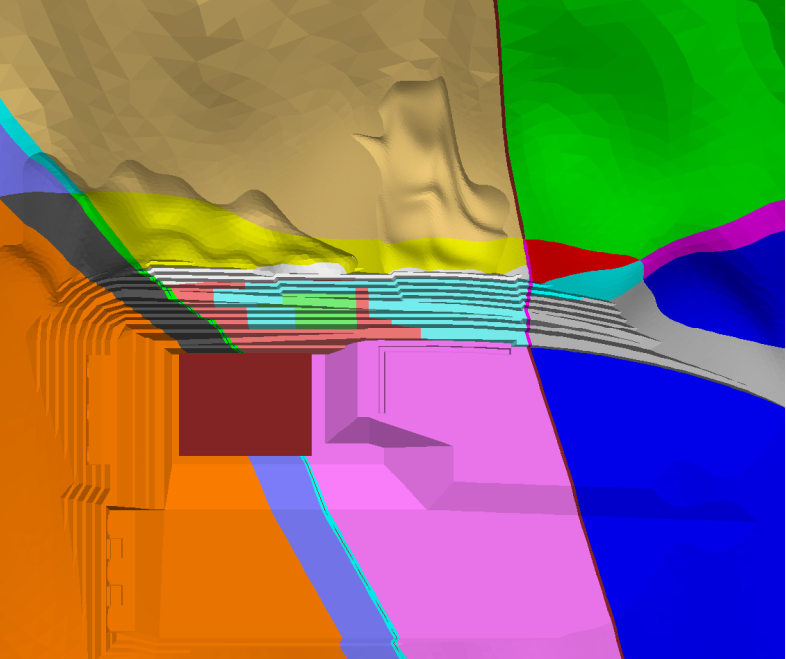

Supplement: Supplementary file 1 — Supplementary Information. [file 41598_2024_57598_MOESM1_ESM.zip › 补充图03.png]

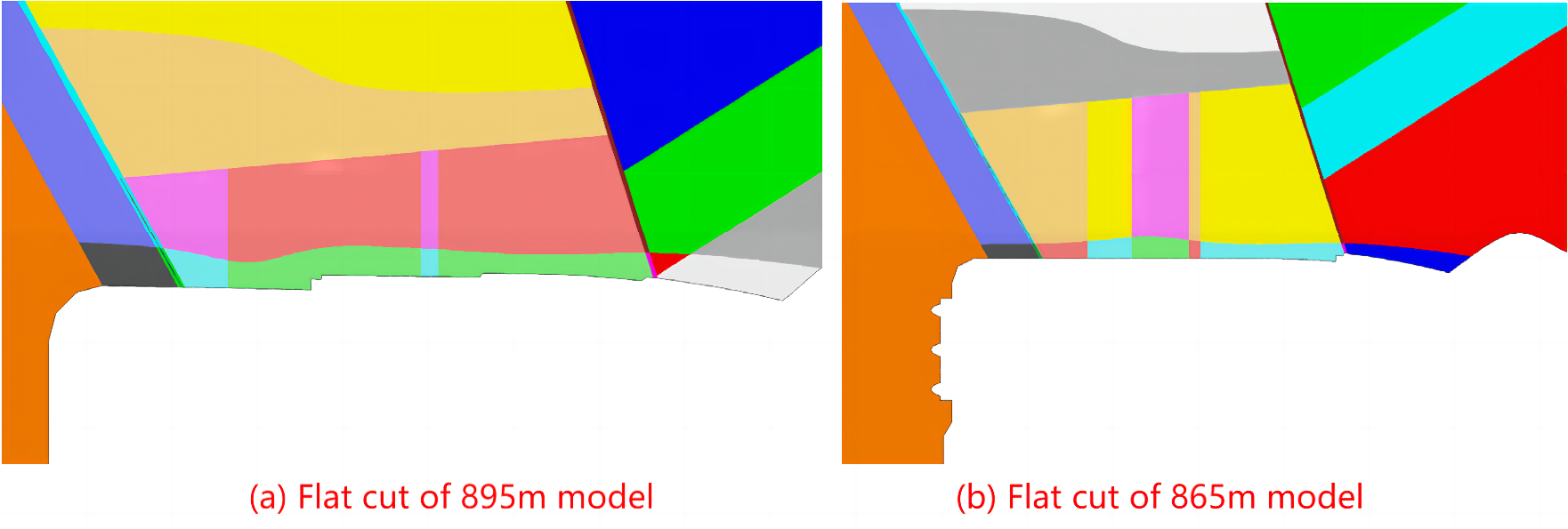

Supplement: Supplementary file 1 — Supplementary Information. [file 41598_2024_57598_MOESM1_ESM.zip › 补充图04(1).png]

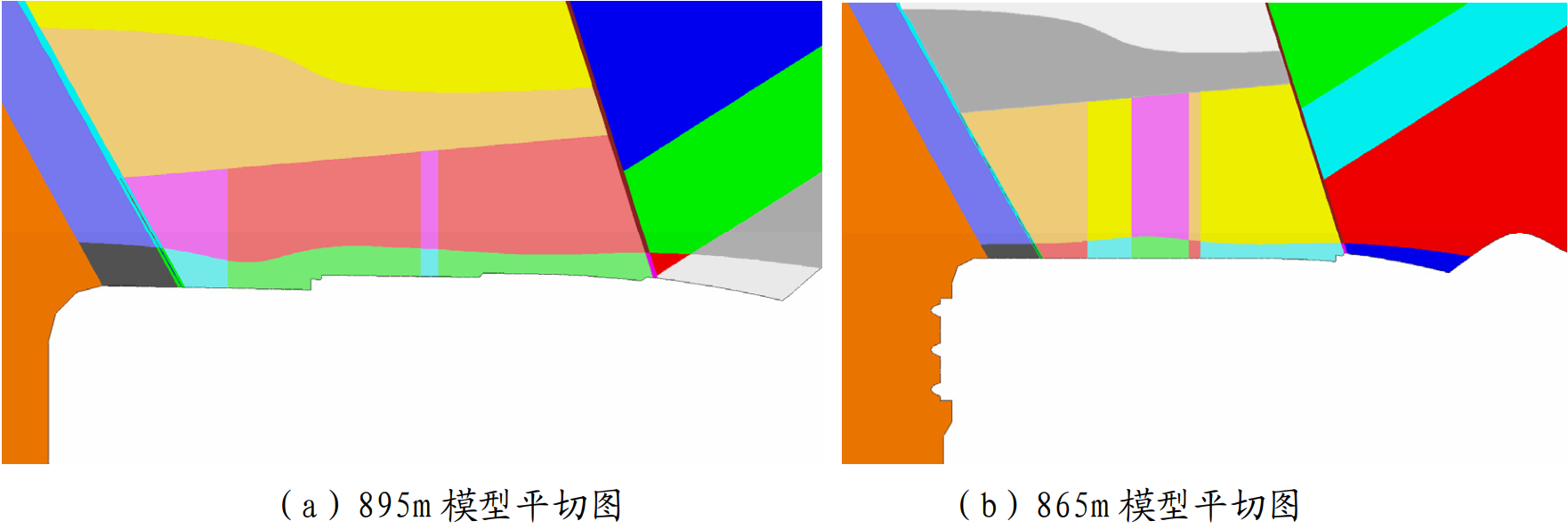

Supplement: Supplementary file 1 — Supplementary Information. [file 41598_2024_57598_MOESM1_ESM.zip › 补充图04.png]

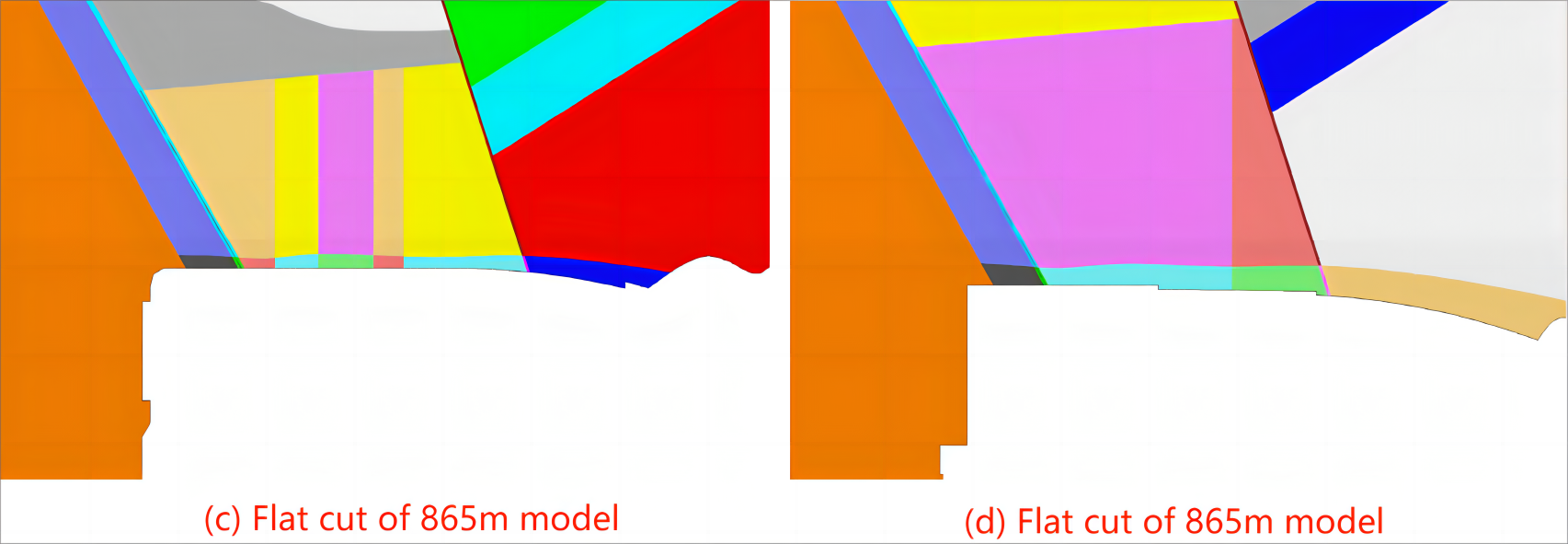

Supplement: Supplementary file 1 — Supplementary Information. [file 41598_2024_57598_MOESM1_ESM.zip › 补充图05(1).png]

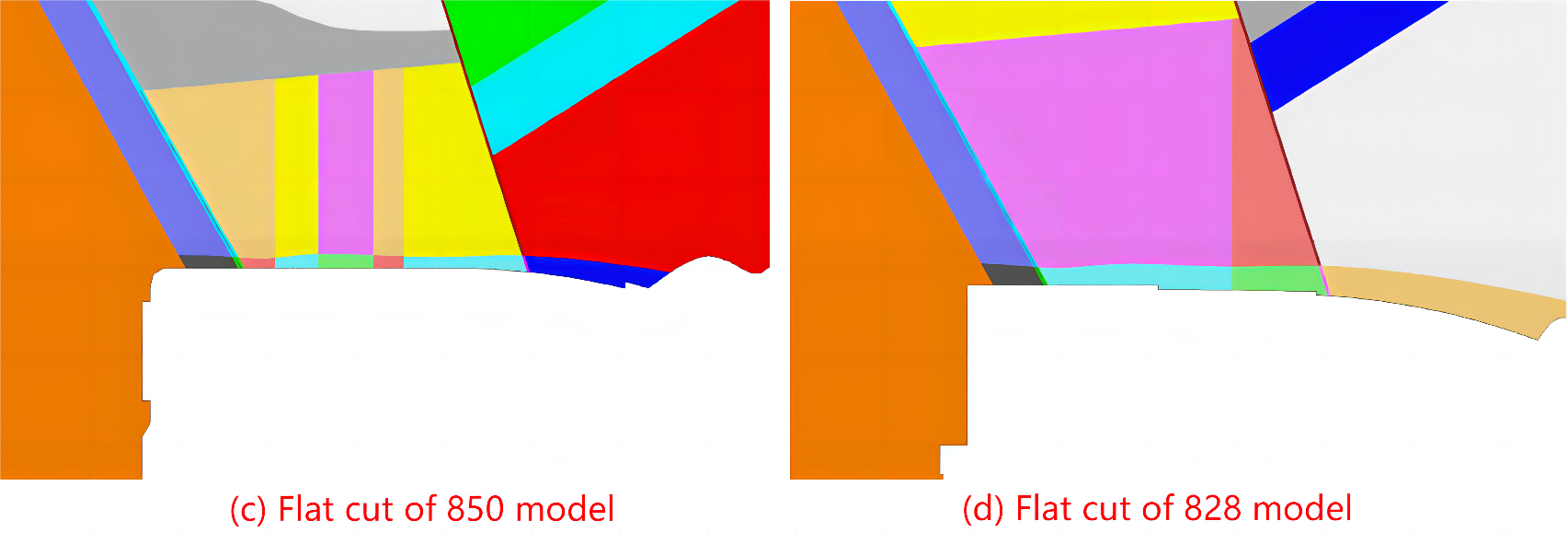

Supplement: Supplementary file 1 — Supplementary Information. [file 41598_2024_57598_MOESM1_ESM.zip › 补充图05(3).png]

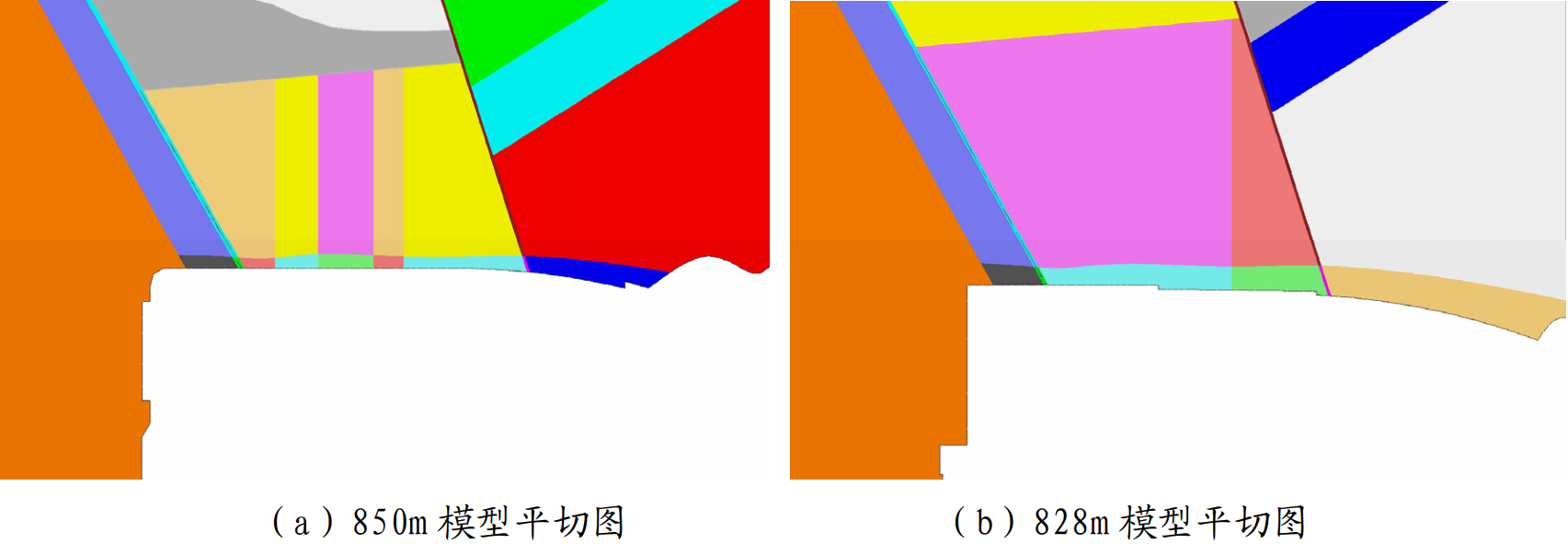

Supplement: Supplementary file 1 — Supplementary Information. [file 41598_2024_57598_MOESM1_ESM.zip › 补充图05.png]
